# Sex-based differences in short- and longer-term diet-induced metabolic heart disease

**DOI:** 10.1152/ajpheart.00467.2023

**Published:** 2024-02-16

**Authors:** Amanda J. Croft, Conagh Kelly, Dongqing Chen, Tatt Jhong Haw, Lohis Balachandran, Lucy A. Murtha, Andrew J. Boyle, Aaron L. Sverdlov, Doan T. M. Ngo

**Affiliations:** ^1^School of Medicine and Public Health, College of Health Medicine and Wellbeing, University of Newcastle, Callaghan, New South Wales, Australia; ^2^Hunter Medical Research Institute, New Lambton Heights, New South Wales, Australia; ^3^School of Biomedical Sciences and Pharmacy, College of Health Medicine and Wellbeing, University of Newcastle, Callaghan, New South Wales, Australia; ^4^Hunter New England Local Health District, Newcastle, New South Wales, Australia

**Keywords:** cardiovascular disease, metabolic heart disease, obesity, RNA methylation, sex differences

## Abstract

Sex-based differences in the development of obesity-induced cardiometabolic dysfunction are well documented, however, the specific mechanisms are not completely understood. Obesity has been linked to dysregulation of the epitranscriptome, but the role of N^6^-methyladenosine (m6A) RNA methylation has not been investigated in relation to the sex differences during obesity-induced cardiac dysfunction. In the current study, male and female C57BL/6J mice were subjected to short- and long-term high-fat/high-sucrose (HFHS) diet to induce obesogenic stress. Cardiac echocardiography showed males developed systolic and diastolic dysfunction after 4 mo of diet, but females maintained normal cardiac function despite both sexes being metabolically dysfunctional. Cardiac m6A machinery gene expression was differentially regulated by duration of HFHS diet in male, but not female mice, and left ventricular ejection fraction correlated with RNA machinery gene levels in a sex- and age-dependent manner. RNA-sequencing of cardiac transcriptome revealed that females, but not males may undergo protective cardiac remodeling early in the course of obesogenic stress. Taken together, our study demonstrates for the first time that cardiac RNA methylation machinery genes are regulated early during obesogenic stress in a sex-dependent manner and may play a role in the sex differences observed in cardiometabolic dysfunction.

**NEW & NOTEWORTHY** Sex differences in obesity-associated cardiomyopathy are well documented but incompletely understood. We show for the first time that RNA methylation machinery genes may be regulated in response to obesogenic diet in a sex- and age-dependent manner and levels may correspond to cardiac systolic function. Our cardiac RNA-seq analysis suggests female, but not male mice may be protected from cardiac dysfunction by a protective cardiac remodeling response early during obesogenic stress.

## INTRODUCTION

It is well recognized that patients with obesity have a higher risk of developing systemic metabolic dysfunction, manifesting as insulin resistance, diabetes, and dyslipidemia (1–3). These obesity-related metabolic abnormalities increase the risk of developing a range of cardiovascular diseases, including metabolic heart disease (MHD) (4–6). MHD, characterized by left ventricular hypertrophy (LVH) and diastolic dysfunction, may progress to heart failure over time (7–9).

There is increased evidence that sex-based differences play an important role in clinical predisposition to obesogenic stress-induced cardiovascular complications (10). Most of the clinical manifestations of obesity-induced cardiovascular and metabolic (cardiometabolic) sequelae show a clear interaction between sex and aging (10, 11). Men tend to develop heart disease 10–15 years earlier than women, who are relatively protected from cardiovascular disease before menopause (12–14). These differences are abolished with advancing age (15).

Previous studies investigating sex-based differences in obesity-induced cardiometabolic dysfunction restricted their investigations to differences in age-related changes in sex hormones as the primary differentiating mechanism(s) (16–18). In addition to the clinical observations that premenopausal women are relatively protected from cardiovascular disease, evidence from rodent models indicates sex hormone deprivation via ovariectomy is sufficient to alter the cardiac function and remodeling (17, 19), and estrogen treatment was protective against myocardial infarction/pressure overload in models of cardiomyopathy (20, 21). Furthermore, while sex-based changes in cardiometabolic functions with shorter- versus longer-term obesogenic diet exposure have previously been investigated, detailed mechanism(s) underpinning these differences are not elucidated.

Epigenetic regulation of genes by interaction with environment contributes to the pathophysiology of obesity and associated cardiometabolic changes (22–26). Considerable progress has been made in the understanding of DNA methylation and histone modifications in cardiovascular and metabolic diseases (27–29), however, investigations of the epitranscriptomic impact on obesity and associated cardiometabolic complications are in their infancy. The most well-known modification of the epitrancriptome, N^6^-methyladenosine (m6A), is added to RNA by “writers”: methyltransferase-like 3 and 14 (METTL3, METTL14); removed by “erasers”: fat mass and obesity-associated protein (FTO) and ALKB homolog 5 (ALKBH5); and interpreted by “readers”: YTH N^6^-methyladenosine RNA-binding protein F 1–3 (YTHDF1-3) and YTH domain containing 1–2 (YTHDC1-2) (30–33). Modification of RNA occurs at a posttranscriptional level and has been reported to influence RNA translation, splicing, transport, and degradation (34–37). The process of m6A modification is reversible and dynamic. The m6A modification has been shown to play an important role in the regulation of lipid accumulation (38–40), diabetes mellitus (41, 42), and obesity (43). Specifically, the dysregulation of m6A patterns, via disruption of demethylase FTO is linked to the pathophysiology of obesity and systemic metabolism (42, 44, 45). Overexpression of FTO increases fat accumulation and induces obesity in mice, whereas knockdown of FTO reduces fat mass and obesity (46, 47). Importantly, FTO expression is decreased in heart failure, with corresponding losses in cardiomyocyte contractile gene transcripts involved in calcium handling and sarcomere dynamics (48). Although there is a paucity of data in the cardiovascular area, a few studies in other systems suggest that there are sex-based differences in the epitranscriptome (49–53). However, to date, sex-based epitranscriptomic changes associated with cardiac complications of obesity have not yet been explored.

Previously, we showed in a mouse model that “Western-style,” high-fat/high-sucrose (HFHS) diet-induced obesity leads to a cardiac phenotype of LVH and diastolic dysfunction similar to that seen in patients with metabolic syndrome (54, 55). In the current study, our goal was to assess sex-based differences in *1*) different durations of obesogenic stress-induced cardiometabolic changes in a mouse model of high-fat/high-sucrose “Western-style” diet feeding, *2*) involvement of posttranscriptional m6A mRNA modification in regulating these cardiometabolic changes, and *3*) cardiac transcriptomic changes and pathways involved in sex-based responses to different durations of obesogenic stress.

## MATERIALS AND METHODS

### Animals

Male and female C57BL/6NJ mice (6–8 wk of age) were randomized to groups (*n* = 7–14) fed either: *1*) a standard chow diet (normal chow NC) containing 4.6% fat/0.0% sucrose, where 12.0% of energy is calculated to be supplied from lipids or *2*) a high-fat/high-sucrose (HFHS) diet containing 36.0% fat/34.6% sucrose, where 59.0% of energy is calculated to be supplied from lipids (SF03-002, Specialty Feeds, Western Australia). Animals were given free access to food and water and maintained on their respective diets for 1 or 4 mo. Animals were housed in groups of four per cage and kept on a standard 12-h:12-h light/dark cycle. Body weights were recorded weekly for the duration of the diet. Animals were supplied by Australian Bioresources, NSW, Australia. All animal experiments were approved by the University of Newcastle Animal Care and Ethics Committee, and conducted in accordance with the Australian Code for the Care and Use of Animals for Scientific Purposes.

### Cardiac Echocardiography

Transthoracic echocardiography examination was performed using a high-frequency ultrasound system (Vevo 1100, Visualsonics Inc., Canada) with a 30-MHz linear probe. Mice were lightly anesthetized with 0.5–2% isoflurane in oxygen, adjusting isoflurane delivery to maintain the heart rate at 500 ± 50 beats/min. To assess left ventricular systolic function, parasternal standard two-dimensional (2-D) long- and short-axis views (LAX and SAX view, respectively) were acquired. Echo analyst was blinded to study groups. Left ventricular ejection fraction, stroke volume, and cardiac output were obtained from the LAX view. For diastolic dysfunction, mitral valve flow was evaluated in the four-chamber apical view using pulsed-wave (PW) Doppler to measure early and late diastolic velocity peak wave (E and A, respectively), expressed as a ratio (E/A). M-mode echocardiographic analysis was used to assess left ventricular interventricular septal dimensions and left ventricular posterior wall thickness. All measurements were averaged over three consecutive cardiac cycles.

### Glucose Tolerance Test

After 1 or 4 mo of feeding on a NC or HFHS diet, fasting glucose levels were measured in mice using an Accuchek Guide glucometer, after overnight fasting. Mice were injected intraperitoneally with 50% glucose at a dose of 2 g/kg. Blood glucose readings were taken at 15, 30, 45, 60, 90, and 120 min postinjection. Glucose readings were plotted over time and area under the curve analysis was carried out using GraphPad Prism 9 software.

### Triglyceride Assay

Triglyceride levels were measured in mouse plasma samples using the Triglyceride Colorimetric Assay kit (Cayman Chemical, 10010303) according to the manufacturer’s protocol. In brief, plasma samples were diluted 1:2 in standard diluent and standards were diluted according to the protocol. Enzyme mixture was added to the samples, which were incubated for 60 min at room temperature before absorbance was measured at 540 nm. Triglyceride concentrations were calculated from the standard curve.

### Plasma Insulin Measurement

Insulin levels were measured in mouse plasma collected at study endpoints after a 3-h starving period, using the Ultrasensitive Mouse Insulin ELISA (Mercodia, 10–1249-01). Plasma samples were assayed according to the manufacturer’s protocol.

### RNA Isolation

RNA was isolated from mouse hearts as follows: 1 mL of Trizol (Invitrogen 15596026) was added to each tissue sample along with a stainless-steel bead. Tissues were disrupted with TissueLyser II (QIAGEN) in 30 s bursts until the tissue was adequately homogenized, resting on ice between bursts. RNA was isolated from the lysate using Isolate II RNA mini kit (Bioline BIO-52073) according to the manufacturer’s protocol. RNA was quantitated by spectrophotometry (NanoDrop 2000, Thermo Fisher Scientific) and quality was assessed using TapeStation analysis for RNA integrity (Agilent).

### Quantitative PCR

cDNA was prepared by reverse transcription of 1 μg of total RNA using High Capacity cDNA Reverse Transcription kit according to the manufacturer’s protocol (Applied Biosystems). Quantitative PCR was carried out using SYBR Green chemistry (Bioline) with gene-specific primers on an ABI QuantStudio 6 Real-Time PCR System. Initial denaturation was carried out at 95°C, 2 min, then 40× cycles of denaturation: 95°C, 5 s; annealing: 60°C, 10 s; and extension: 72°C, 30 s were carried out for amplification. Melting curves were carried out to ensure amplification of single products. Peptidylprolyl isomerase A (*Ppia*) was used as the housekeeper for normalization. Primer sequences and amplicon sizes were as follows: *Mettl3,* NM_019721 (F: AGGACTCTGGGCACTTGGATT; R: GCAGGTGCATCTGGCGTAG; Amplicon size: 250 bp); *Mettl14*, NM_201638 (F: GGACCTTGGGAGAGTATGCTT; R: TCACGGTTCCTTTGATCCCC; Amplicon size: 167 bp); *Alkbh5*, NM_172943 (F: GTGACTGTGCTCAGTGGGTA; R: TCCAATCGCGGTGCATCTAA; Amplicon size: 122 bp); *Ppia*, NM_008907 (F: CATTCCTGGACCCAAAACG; R: GGCAAATGCTGGACCAAAC, Amplicon size: 149 bp). Primers designed in-house were subject to primer BLAST to ensure the absence of nonspecific amplicons. Sigma Kicqstart predesigned primer sets were used for *Fto*, NM_011936 (M_Fto_1), *Ythdf1*, NM_173761 (M_Ythdf1_1) and *Ythdf2*, NM_145393 (M_Ythdf2_1).

### RNA Sequencing

RNA-sequencing libraries were generated from 1 μg total RNA using the Illumina Stranded mRNA kit as per the manufacturer’s instructions. Libraries were sequenced using Illumina NovaSeq6000 with v1.5 chemistry and 150-bp paired-end reads to a minimum depth of 20 million reads. Normalization and differential gene expression analysis were conducted using the edgeR (version 3.38.4) through the R 4.2.2 package. Pair-wise comparisons were conducted between diets according to sex and diet duration (Male 1 mo NC vs. HFHS; Male 4 mo NC vs. HFHS; Female 1 mo NC vs. HFHS; Female 4 mo NC vs. HFHS); *n* = 3 per group. Pair-wise comparisons between sexes in the absence of diet were also conducted for each time point. Genes with absolute value log2 fold change >0.58 and *P* value <0.05 were considered to be differentially expressed. InteractiVenn was used to analyze the overlap between differential gene expression using Venn diagrams (56). Analysis was conducted using the ShinyGO 0.77 Web-based tool to identify GO Biological Processes and KEGG pathways enriched in our datasets (57, 58). Pathways with a false-discovery rate <0.05 were considered to be significantly enriched. Data were also analyzed with GSEA v4.3.2 using the Gene Ontology: Biological Processes and Canonical Pathways databases (59–61). Protein interaction networks were generated using the STRING database (62).

### Statistical Analysis

Statistical analysis was conducted using GraphPad Prism version 9. Data are expressed as mean ± standard error of the mean. Two-way ANOVA was used where two or more groups were compared, followed by post hoc analysis with Tukey’s test. Pairwise comparisons were analyzed with unpaired *t* tests. *P* values of less than 0.05 were considered statistically significant.

## RESULTS

### Cardiometabolic Assessment of Male and Female Mice Subjected to Short- and Long-Term High-Fat, High-Sucrose Feeding

**Figure 1.**
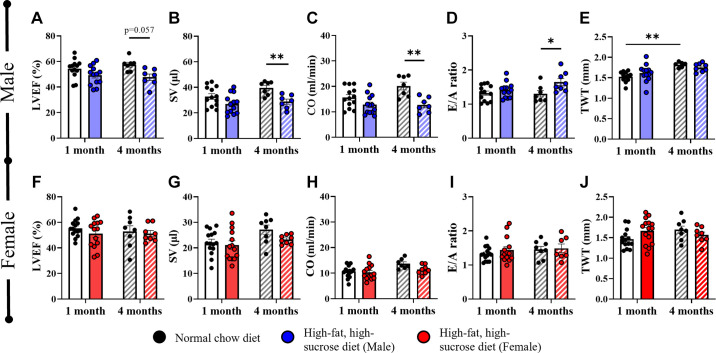
Echocardiographic analysis of the cardiac function of male and female mice after 1- and 4-mo high-fat/high-sucrose diet. Male mice: *A*: percentage left ventricular ejection fraction (LVEF). *B*: stroke volume (SV). *C*: cardiac output (CO). *D*: E/A ratio. *E*: total wall thickness (TWT). Female mice: *F*: percentage LVEF. *G*: SV. *H*: CO. *I*: E/A ratio. *J*: TWT. For statistical analysis, two-way ANOVAs with post hoc Tukey tests were performed. All values are represented as means with error bars representing standard error of the mean. *P < 0.05, **P < 0.01. *n* = 7–14 mice/group, as specified by number of data points in each graph.

To assess any differences in cardiac functional effects according to the duration of the HFHS diet in male and female mice, cardiac echocardiography was carried out after 1 and 4 mo of feeding. After 1 mo of HFHS diet, there were no changes in left ventricular ejection fraction (LVEF, *P* = 0.24), stroke volume (SV, *P* = 0.16), cardiac output (CO, *P* = 0.23), or E/A ratio (*P* = 0.80) in male mice; however, after 4 mo, males showed a trend toward reduction in LVEF (*P* = 0.06), significant reductions in SV (*P* < 0.05) and CO (*P* < 0.01), and significantly increased E/A ratio (*P* < 0.05) (Fig. 1, *A*–*D*). Total wall thickness was unchanged in male mice after 1 or 4 mo of HFHS diet (Fig. 1*E*, 1 mo: *P* = 0.48; 4 mo: *P* = 0.83). Similarly to males, female mice showed no difference in LVEF (*P* = 0.60), SV (*P* = 0.94), CO (*P* = 0.97), or E/A ratio (*P* = 0.75) after 1 mo of HFHS feeding; however, unlike males, female mice did not develop cardiac functional changes after 4 mo HFHS feeding either with respect to LVEF (*P* = 0.98), SV (*P* = 0.43), CO (*P* = 0.27), or E/A ratio (*P* = 0.99) (Fig. 1, *F–I*). Total wall thickness showed no changes after 1 or 4 mo of diet in female mice (Fig. 1*J*, 1 mo: *P* = 0.18; 4 mo: *P* = 0.77). Diet had a significant effect on LVEF, SV, CO, and E/A ratio in male mice, but not in female mice (two-way ANOVA: LVEF, Males: *P* < 0.01; Females: *P* = 0.31; SV, Males: *P* < 0.01; Females: *P* = 0.13; CO, Males: *P* < 0.001; Females: *P* = 0.09; E/A ratio, Males: *P* < 0.05; Females: *P* = 0.45). Age had a significant effect on SV and CO in female but not in male mice (SV, Males: *P* = 0.08; Females: *P* < 0.05; CO, Males: *P* = 0.09; Females: *P* < 0.05). Conversely, age had a significant effect on total wall thickness in males but not in females (Males: *P* < 0.001; Females: *P* = 0.43). There were no significant interactions between age and diet for any of the parameters tested.

**Figure 2.**
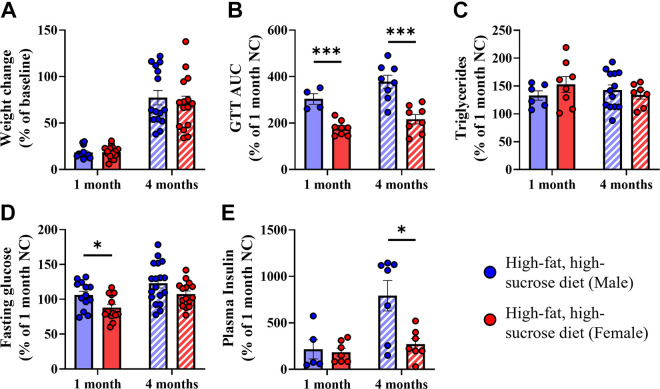
Body weight and systemic metabolic parameters in male and female mice after 1- and 4-mo high-fat/high-sucrose (HFHS) diet. *A*: percentage weight change compared with baseline weight measurements. *B*: glucose tolerance test (GTT), area under curve (AUC) analysis. *C*: plasma triglycerides. *D*: fasting glucose. *E*: plasma insulin. *B–E* are expressed as mean percentage change of HFHS diet groups compared with sex-matched, normal chow diet (1 mo exposure) groups, with error bars representing standard error of the mean. For statistical analysis, unpaired *t* tests performed between male and female groups at each time point. *P < 0.05, ***P < 0.001. *n* = 4–14 mice/group, as specified by number of data points in each graph.

Mice body weights and systemic metabolic functions are summarized in Fig. 2 and Table 1. Despite the sex differences we observed in cardiac function, male and female mice gained a similar amount of weight from baseline (Fig. 2*A*). Glucose tolerance testing showed significant dysfunction in both male and female mice after 1 and 4 mo of HFHS feeding versus NC diet (Table 1), however, males were proportionately worse than females at both time points (Fig. 2*B*; Males vs. Females, 1 mo: *P* < 0.001; 4 mo: *P* < 0.001). Female mice had significantly increased triglyceride levels after 1 mo and 4 mo of feeding, whereas males were significantly increased at 4 mo only (Table 1), however, the percentage change compared with the 1-mo NC groups (matched for sex) was not significantly different between males and females (Fig. 2*C*; Males vs. Females, 1 mo: *P* = 0.29; 4 mo: *P* = 0.55). Male and female mice showed increased fasting glucose levels only after 4 mo of HFHS feeding versus NC diet (Table 1), but males had incrementally higher levels than females after 1 mo of HFHS diet only (Fig. 2*D*; Males vs. Females, 1 mo: *P* < 0.05; 4 mo: *P* = 0.08). Finally, plasma insulin levels were increased in males only after 4 mo of HFHS diet versus NC (Table 1) and were proportionately higher than in female mice after 4 mo of diet (Fig. 2*E*; Males vs. Females, 1 mo: *P* = 0.76; 4 mo: *P* < 0.05).

**Table 1.** Body weight and metabolic parameters of male and female mice fed high-fat, high-sucrose diet for 1 or 4 mo

	Male							Female						
	1-Mo diet consumption		4-Mo diet consumption		Two-Way ANOVA			1-Mo diet consumption		4-Mo diet consumption		Two-Way ANOVA		
	NC	HFHS	NC	HFHS	Diet	Time	Interaction	NC	HFHS	NC	HFHS	Diet	Time	Interaction
% BW change	11.2 ± 1.0	18.3 ± 1.8	**30.7 ± 3.1#**	77.3 ± 7.8****####	<0.0001	<0.0001	<0.0001	12.6 ± 1.6	18.7 ± 1.7	**30.0 ± 2.5#**	71.4 ± 7.6****####	<0.00	<0.00	0.0004
GTT, AUC	513.8 ± 22.7	1559.5 ± 117.6***	**1159.6 ± 69.4#**	1940.8 ± 142.6***	<0.0001	0.0012	NS	792.0 ± 116.2	1414.3 ± 89.1**	**1680.3 ± 112.4###**	1717.1 ± 162.5	0.0137	<0.00	0.0269
TG, mg/dL	49.9 ± 5.8	66.4 ± 4.2	44.4 ± 3.2	71.3 ± 4.6***	<0.0001	NS	NS	37.1 ± 2.4	56.7 ± 5.3**	33.8 ± 3.8	49.8 ± 2.9*	0.0001	NS	NS
Glucose, mmol/L	7.6 ± 0.3	8.0 ± 0.4	7.6 ± 0.3	9.3 ± 0.5*	0.0132	NS	NS	7.6 ± 0.3	6.7 ± 0.4	7.3 ± 0.3	**8.2 ± 0.4##**	NS	NS	0.0072
Insulin, ng/mL	0.5 ± 0.1	1.1 ± 0.5	1.5 ± 0.3	4.1 ± 0.8*##	0.0131	0.0031	NS	0.2 ± 0.1	0.4 ± 0.1	0.4 ± 0.1	0.6 ± 0.1	0.0322	NS	NS

Values are means ± SE. AUC, area under curve; BW, body weight; GTT, glucose tolerance test; HFHS, high-fat, high-sucrose diet; NC, normal chow diet; TG, triglycerides. *P < 0.05, **P < 0.01, ***P < 0.001, ****P < 0.0001, vs. age-matched NC. #*P* < 0.05*, ##P* < 0.01*, ###P* < 0.001*, ####P* < 0.0001, vs. 1 mo time point in the same diet. Boldface indicates statistical significance. NS, not significant. *n* = 4–14 mice/group.

### Sex Differences in Cardiac Expression of RNA Methylation Machinery Genes in Response to High-Fat, High-Sucrose Feeding

**Figure. 3.**
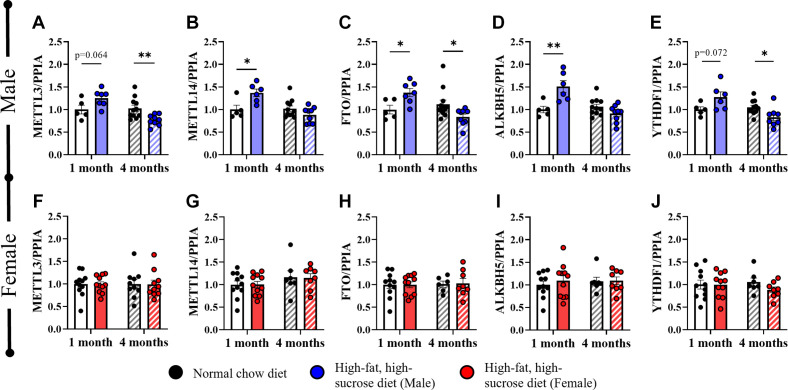
Cardiac mRNA gene expression analysis of m6A machinery genes in male and female mice after 1- and 4-mo high-fat/high-sucrose (HFHS) diet. Male mice: *A*: *Mettl3*. *B*: *Mettl14*. *C*: *Fto*. *D*: *Alkbh5*. *E*: *Ythdf1*. Female mice: *F*: *Mettl3*. *G*: *Mettl14*. *H*: *Fto*. *I*: *Alkbh5*. *J*: *Ythdf1*. Data are gene expression levels normalized by *Ppia* levels, relative to age- and sex-matched normal chow diet group, expressed as means with error bars representing standard error of the mean. For statistical analysis, unpaired *t* tests were performed between NC and HFHS diet groups at each time point. *P < 0.05, **P < 0.01. *n* = 5–10 mice/group, as specified by number of data points in each graph.

To assess possible involvement of RNA methylation machinery in regulating these sex-based cardiometabolic differences, we examined cardiac expression levels of RNA methylation machinery genes. These genes were differentially regulated in male mice fed HFHS diet at 1 mo and 4 mo while they were not altered in female mice, irrespective of duration of HFHS diet feeding. There was a general upregulation of all m6A gene regulators in male mice fed HFHS diet at 1 mo and a general downregulation at 4 mo for writers *Mettl3* (Fig. 3*A*, 1 mo: *P* = 0.064; 4 mo: *P* = 0.007), *Mettl14* (Fig. 3*B*, 1 mo: *P* = 0.03; 4 mo: 0.12); erasers *Fto* (Fig. 3*C*, 1 mo: *P* = 0.03; 4 mo: *P* = 0.02), *Alkbh5* (Fig. 3*D*, 1 mo: *P* = 0.006; 4 mo: *P* = 0.09); and reader *Ythdf1* (Fig. 3*E*, 1 mo: *P* = 0.04; 4 mo: *P* = 0.02). Female mice showed no changes in expression of these genes at either time point (Fig. 3, *F–J*, *P* = NS).

### Relationship between m6A Machinery Gene Expression and Cardiac Function

**Figure 4.**
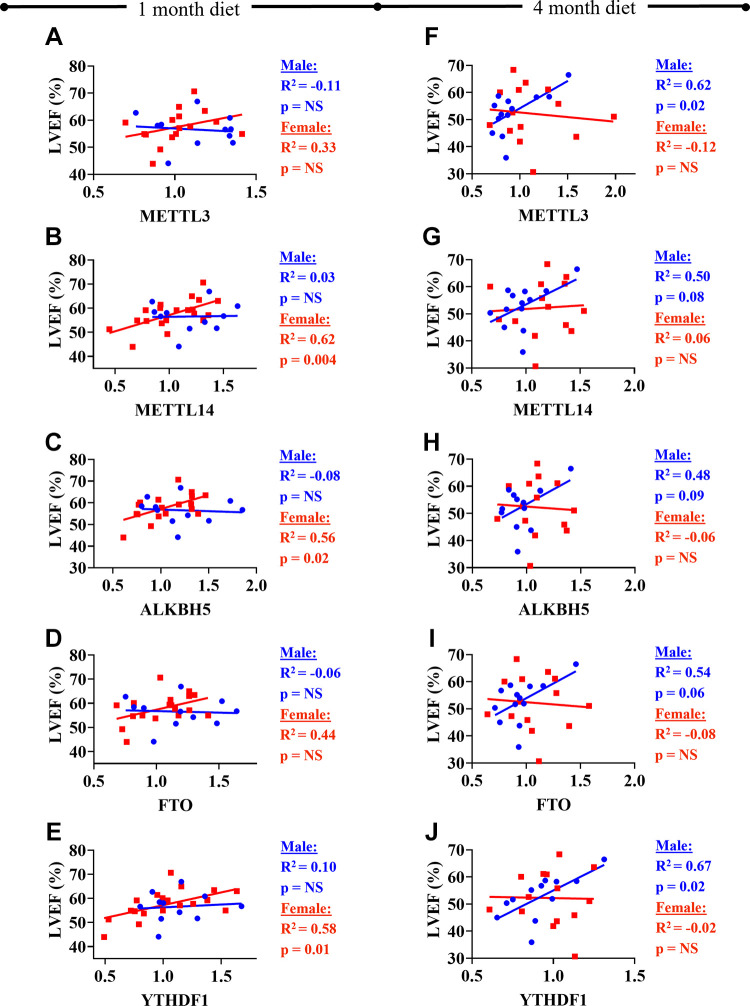
Regression analysis of left ventricular ejection fraction versus m6A machinery gene expression levels after 1- and 4-mo of high-fat/high-sucrose diet. 1 mo diet: *A*: left ventricular ejection fraction (LVEF) versus *Mettl3*. *B*: LVEF versus *Mettl14*. *C*: LVEF versus *Alkbh5*. *D*: LVEF versus *Fto*. *E*: LVEF versus *Ythdf1*. 4 mo diet: *F*: LVEF versus *Mettl3*. *G*: LVEF versus *Mettl14*. *H*: LVEF versus *Alkbh5*. *I*: LVEF versus *Fto*. *J*: LVEF versus *Ythdf1*. Blue circles, blue regression line: Male mice; red squares, red regression line: Female mice. For statistical analysis, linear regression analysis was performed overall and for males and females separately. *n* = 11–14 mice/group, as specified by number of data points in each graph.

To assess whether expression levels of m6A machinery genes are associated with cardiac function, we correlated gene expression levels at each time point against corresponding LVEF (Fig. 4). In general, a number of m6A methylation machinery genes significantly correlated with LVEF, predominantly driven by female mice groups at 1 mo time point: *Mettl14* (R = 0.62, *P* = 0.004), *Alkbh5* (R = 0.56, *P* = 0.02), and *Ythdf1* (R = 0.58, *P* = 0.01). However, the relationship of m6A RNA machinery genes and LVEF was transformed at 4 mo, with male mice group driving this relationship: *Mettl3* (R = 0.62, *P* = 0.02), *Mettl14* (R = 0.50, *P* = 0.08), *Alkbh5* (R = 0.48, *P* = 0.09), *Fto* (R = 0.54, *P* = 0.06), and *Ythdf1* (R = 0.674, *P* = 0.02).

### Sex Differences in Cardiac Transcriptome in Response to Short- and Long-Term High-Fat, High-Sucrose Feeding

**Figure 5.**
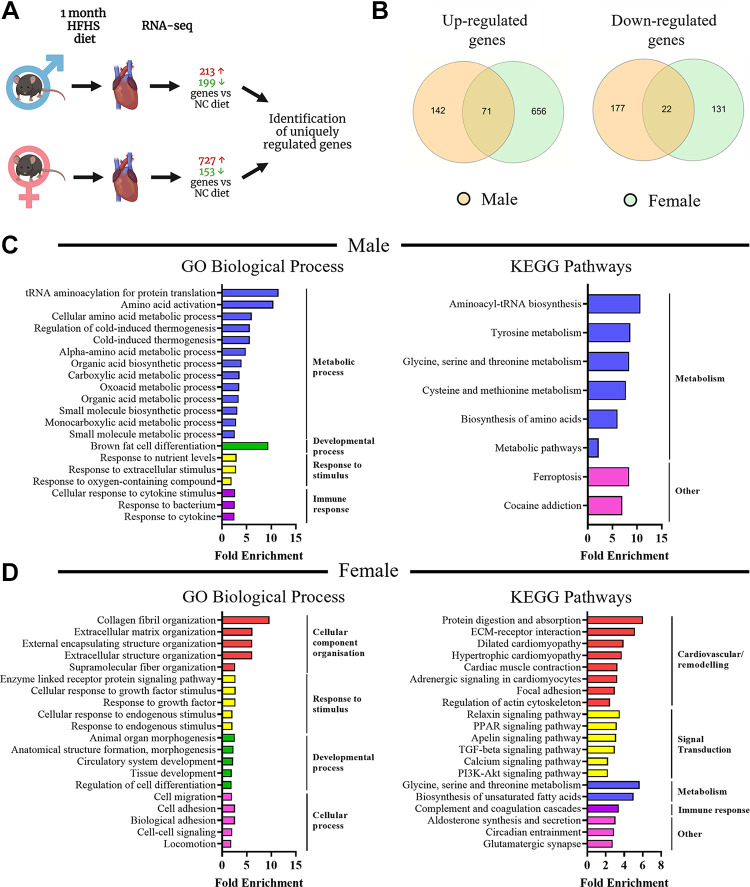
RNA-sequencing analysis of cardiac transcriptome in male and female mice after 1 mo of high-fat/high-sucrose diet, *n* = 3 mice/group. *A*: overview of RNA-seq analysis in male and female mouse hearts. Generated using Biorender.com. *B*: Venn diagram showing commonly and uniquely differentially up- and downregulated genes in male and female mice in response to 1 mo high-fat/high-sucrose diet. *C*: gene enrichment analysis in genes uniquely up- and downregulated in male mice in response to high-fat/high-sucrose diet. *D*: gene enrichment analysis in genes uniquely up- and downregulated in female mice in response to high-fat/high-sucrose diet. For each analysis, the top 20 results that scored FDR <0.05 are displayed.

**Figure 6.**
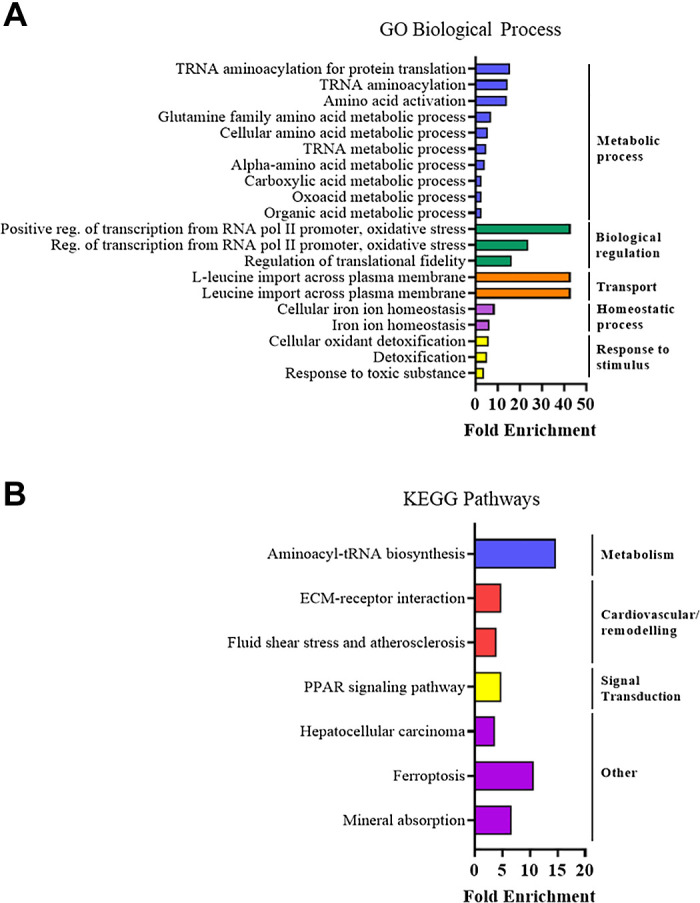
Enrichment analysis on differentially expressed genes between male and female mice in the absence of high-fat/high-sucrose (HFHS) diet at 1 mo time point. *A*: Gene ontology, biological processes. *B*: KEGG pathway analysis. For each analysis, the top 20 results that scored false discovery rate (FDR) < 0.05 are displayed. *n* = 3 mice/group.

To investigate further potential mechanisms involved in the sex differences we observed in cardiac function, RNA-sequencing analysis was performed in cardiac tissues after different durations of HFHS diet. First, we observed that the cardiac gene expression response after 1 mo of HFHS diet was vastly different in magnitude between the sexes, with male mice displaying a total of 412 (213↑ + 199↓) differentially expressed genes and females showing 880 (727↑ + 153↓) (Fig. 5*A*). We compared the gene lists to find commonly and uniquely upregulated and downregulated genes in male versus female mice after short-term HFHS diet (Fig. 5*B*). Intriguingly, we identified that the majority of differentially expressed genes were unique to sex: male mice showed 141/213 genes uniquely upregulated and 175/199 genes uniquely downregulated and female mice showed 654/727 genes uniquely upregulated and 130/153 genes uniquely downregulated. To investigate biological processes and signaling pathways differentially regulated in the heart after short-term feeding, we used two different approaches (ShinyGO and GSEA) to conduct enrichment analyses on genes uniquely regulated in male or female mice. In male mice, metabolic processes and pathways were the most significantly represented as shown by ShinyGO analysis of GO Biological Processes and KEGG pathway databases (Fig. 5*C*, Tables 2 and 3). Metabolic-related pathways were also identified using GSEA analysis of GO Biological Processes and Canonical Pathways (Tables 4 and 5). In female mice, the list of highly represented GO Biological Processes included those related to extracellular matrix organization, response to stimuli, and developmental processes and highly represented KEGG pathways included those related to cardiovascular disease, cardiac function, remodeling, and PPAR, TGFβ, calcium, and PI3K-Akt signal transduction pathways (Fig. 5*D*, Tables 6 and 7), which was in agreement with the GSEA analyses (Tables 8 and 9). We also assessed direct differences in cardiac gene expression between males and females in the absence of HFHS diet and found that there were 119 genes upregulated and 283 genes downregulated in females versus males at this time point. Analysis of GO Biological Processes (Fig. 6*A*) showed that there were differences in enrichment of metabolic, biological regulation and transport processes, and KEGG pathway analysis (Fig. 6*B*) showed differences in metabolic, ECM remodeling, and PPAR signaling pathways.

**Table 2.** ShinyGO Gene Ontology Biological Processes analysis showing up- and downregulated genes in male cardiac tissues in response to 1-mo HFHS feeding

Pathway	Genes (*n*)	Pathway Genes (*n*)	Fold Enrichment	Enrichment FDR	Upregulated Genes (vs. Normal Chow Diet)	Downregulated Genes (vs. Normal Chow Diet)
Oxoacid metabolic process	47	905	3.5	1.60E-10	Pck1 Adipoq Prkar2b Cdo1 Scd1 Ces1f Cyp2e1 Elovl6 Aspg Bcat1 Gpd1 Brca1 Aacs Aspa Dct Dbi Insig1 Pfkfb2 Abcd2 Slc27a3 Ech1 Decr1	Mars1 Atf4 Cars Mid1ip1 Iars Yars Ddc Gatm Eprs Sars Gars Gclm Hoga1 Al dh1l2 Cth Gsta1 Aldh18a1 Nr5a2 Upb1 Psat1 Nr1h4 Pycr1 Phgdh Trib3 Asns
Carboxylic acid metabolic process	47	893	3.5	1.60E-10	Pck1 Adipoq Prkar2b Cdo1 Scd1 Ces1f Cyp2e1 Elovl6 Aspg Bcat1 Gpd1 Brca1 Aacs Aspa Dct Dbi Insig1 Pfkfb2 Abcd2 Slc27a3 Ech1 Decr1	Mars1 Atf4 Cars Mid1ip1 Iars Yars Ddc Gatm Eprs Sars Gars Gclm Hoga1 Aldh1l2 Cth Gsta1 Aldh18a1 Nr5a2 Upb1 Psat1 Nr1h4 Pycr1 Phgdh Trib3 Asns
Organic acid metabolic process	47	937	3.4	3.75E-10	Pck1 Adipoq Prkar2b Cdo1 Scd1 Ces1f Cyp2e1 Elovl6 Aspg Bcat1 Gpd1 Brca1 Aacs Aspa Dct Dbi Insig1 Pfkfb2 Abcd2 Slc27a3 Ech1 Decr1	Mars1 Atf4 Cars Mid1ip1 Iars Yars Ddc Gatm Eprs Sars Gars Gclm Hog a1 Aldh1l2 Cth Gsta1 Aldh18a1 Nr5a2 Upb1 Psat1 Nr1h4 Pycr1 Phgdh Trib3 Asns
Small molecule metabolic process	66	1,703	2.6	4.75E-10	Pck1 Adipoq Car3 Prkar2b Cdo1 Scd1 Ces1f Cyp2e1 Elovl6 Aspg Car13 Cbr2 Car5b Degs2 Bcat1 Gpd1 Cebpa Dbh Sqle Brca1 Aacs Aspa Ces2e Sik1 Gpd2 Pfkfb3 Dct Dbi Insig1 Pfkfb2 Abcd2 Ppp1r3c Mme Slc2 7a3 Ech1 Ip6k3 Decr1	Mars1 Atf4 Cars Mid1ip1 Dhrs13 Iars Yars Ddc Gatm Eprs Sars Dnph1 Gars Gclm Hoga1 Aldh112 Cth Ttpa Gsta1 Idh18a1 Nr5a2 Upb1 Psat1 Nr1h4 Pycr1 Phgdh Trib3 Asns Mthfd2
Cellular amino acid metabolic process	23	256	6	5.38E-09	Cdo1 Aspg Bcat1 Dct	Mars1 Atf4 Cars Iars Yars Ddc Eprs Sars Gars Gclm Hoga1 Cth Aldh18a1 Upb1 Psat1 Nr1h4 Pycr1 Phgdh Asns
Response to cytokine	35	922	2.5	2.85E-04	Pck1 Adipoq Krt8 Acp5 Sh2b2 Gpd1 Cebpa Serpina3c Brca1	Bbs4 Ifi47 Gbp5 Nlrc5 Gbp4 Eprs Tgtp2 Cd274 Iigp1 Gclm Tgtp1 Btk Cth Gm12185 Ppbp Nr5a2 Nr1h4
Cellular response to cytokine stimulus	32	811	2.6	3.01E-04	Ackr4 Smpd3 Tfrc I127ra Cebpb Mcm2 Il1rl2 Mme Hyal	Bbs4 Ifi47 Gbp5 Nlrc5 Gbp4 Eprs Tgtp2 Iigp1 Gclm Tgtp1 Btk Cth Gm12185 Ppbp Nr5a2 Nr1h4
Small molecule biosynthetic process	25	536	3.1	3.01E-04	Pck1 Adipoq Krt8 Sh2b2 Gpd1 Cebpa Brca1 Ackr4 Smpd3 Tfrc Il27ra Cebpb Mcm2 Il1rl2 Mme Hyal1	Atf4 Mid1ip1 Gatm Hoga1 Cth Aldh18a1 Upb1 Psat1 Pycr1 Phgdh Trib3 Asns
Monocarboxylic acid metabolic process	26	610	2.9	7.16E-04	Pck1 Adipoq Cdo1 Scd1 Elovl6 Bcat1 Gpd1 Dbh Brca1 Sik1 Gpd2 Insig1 Ip6k3	Mid1ip1 Gatm Hoga1 Gsta1 Nr5a2 Nr1h4 Phgdh Trib3
Organic acid biosynthetic process	17	287	4	7.16E-04	Pck1 Adipoq Prkar2b Scd1 Ces1f Cyp2e1 Elovl6 Gpd1 Brca1 Aacs Aspa Dbi Insig1 Pfkfb2 Abcd2 Slc27a3 Ech1 Decr1	Mid1ip1 Gatm Hoga1 Cth Aldh18a1 Upb1 Psat1 Pycr1 Phgdh Trib3 Asns
Brown fat cell differentiation	8	57	9.4	7.23E-04	Cdo1 Scd1 Elovl6 Bcat1 Brca1 Insig1	
TRNA amino-acylation for protein translation	7	41	11.5	7.55E-04	Ucp1 Adipoq Scd1 Dio2 Sh2b2 Adig Cebpa Cebpb	Mars1 Cars Iars Yars Eprs Sars Gars
Response to bacterium	29	745	2.6	8.25E-04		Gbp5 Nlrc5 Gbp4 Tgtp2 H2bc6 Cd274 Iigp1 Tgtp1 Naaladl2 Gsta1 Ppbp Gsta2 Nr1h4
Response to oxygen-containing compound	52	1,798	1.9	8.25E-04	Pck1 Adipoq Car3 Hp Scd1 Cyp2e1 Acp5 Car5b Pyg1 Cnr2 Alp1 Baiap2 Il27ra Cebpb Thrsp Amy1	Sybu Mars1 Atf4 Cts1 Gatm Eprs Cldn5 Cd274 Gclm Cacnb1 Cdk5r1 Btk Hmox1 Ppbp Nr1h4 Pycr1 Gdf15 Tr ib3 Asns
Response to extracellular stimulus	24	560	2.9	8.52E-04	Ucp1 Pck1 Adipoq Car3 Hp Cdo1 Scd1 Krt8 Cyp2e1 Cib2 Aldh3a1 Acp5 Grin3a Ntrk3 Sh2b2 Npas4 Kcnc2 Gpd1 F7 Dbh Serpina3c Brca1 Aacs Smpd3 Cnr2 Alp1 Insig1 Baiap2 Gcnt1 Cebpb Pfkfb2 Ccnd1 Hyal1	B bs4 Mars1 Atf4 Ctsl Gatm BC048403 Gclm Ttpa Hmox1 Nr1h4 Gdf15 Asns
Response to nutrient levels	23	524	3	8.52E-04	Ucp1 Pck1 Adipoq Aldh3a1 F7 Nupr1l Aacs Sik1 Tfrc Alpl Ccnd1 Slc25a25	Bbs4 Mars1 Atf4 Ctsl Gatm BC048403 Gclm Ttpa Hmox1 Nr1h4 Gdf15 Asns
α-Amino acid metabolic process	13	181	4.8	8.52E-04	Ucp1 Pck1 Adipoq Aldh3a1 F7 Nupr1l Aacs Sik1 Alpl Ccnd1 Slc25a25	Atf4 Gclm Hoga1 Cth Aldh18a1 Psat1 Nr1h4 Pycr1 Phgdh Asns
Cold-induced thermogenesis	11	131	5.6	8.52E-04	Cdo1 Aspg Dct	Atf4 Gatm
Regulation of cold-induced thermogenesis	11	131	5.6	8.52E-04	Ucp1 Adipoq Scd1 Elovl6 Dio2 Cidea Dbh Cebpb Decr1	Atf4 Gatm
Amino acid activation	7	45	10.4	8.52E-04	Ucp1 Adipoq Scd1 Elovl6 Dio2 Cidea Dbh Cebpb Decr1	Mars1 Cars Iars Yars Eprs Sars Gars

Genes (*n*) refers to number of differentially expressed genes in the specified pathway. Pathway genes (*n*) refers to the total number of genes in the specified pathway. FDR, false discovery rate; HFHS, high-fat, high-sucrose. *n* = 3 mice per group.

**Table 3.** ShinyGO KEGG pathway analysis showing up- and downregulated genes in male cardiac tissues in response to 1-mo HFHS feeding

Pathway	Genes (*n*)	Pathway Genes (*n*)	Fold Enrichment	Enrichment FDR	Upregulated Genes (vs. Normal Chow Diet)	Downregulated Genes (vs. Normal Chow Diet)
Metabolic pathways	53	1,597	2.2	7.47E-06	Dbh Acp5 Hyal1 Aldh3a1 Aoc3 Aspa Pygl Degs2 Dct Sqle Cbr2 Cyp2e1 Pfkfb2 Pfkfb3 Pck1 Car13 Car3 Alpl Bcat1 Cyp2b10 Aacs Pde3b Car5b Smpd3 Nnmt Cdo1 Scd1 Gcnt1 Elovl6 Mgat5b Cox6b2 Setdb2 Amy1	Hmox1 Mthfd2 Dd c Aldh1l2 Psat1 Aldh18a1 Pycr1 Hoga1 Eprs Gatm Gclm Cth Extl1 Asns Upb1 Mars1 Rimkla Phgdh Gsta2 Gsta1
Aminoacyl-tRNA biosynthesis	7	44	10.7	4.50E-04		Cars Eprs Yars Gars Iars Mars1 Sars
Cysteine and methionine metabolism	6	52	7.8	9.82E-03	Bcat1 Cdo1	Psat1 Gclm Cth Phgdh
Biosynthesis of amino acids	7	78	6	1.01E-02	Bcat1	Psat1 Aldh18a1 Pycr1 Cth Asns Phgdh
Ferroptosis	5	40	8.4	1.08E-02	Tfrc	Hmox1 Slc3a2 Slc40a1 Gclm
Glycine, serine and threonine metabolism	5	40	8.4	1.08E-02	Aoc3	Psat1 Gatm Cth Phgdh
Tyrosine metabolism	5	39	8.6	1.08E-02	Dbh Aldh3a1 Aoc3 Dct	Ddc
Cocaine addiction	5	48	7	2.22E-02	Grin2c Grin3a	Ddc Atf4 Cdk5r1

Genes (*n*) refers to number of differentially expressed genes in the specified pathway. Pathway genes (*n*) refers to the total number of genes in the specified pathway. FDR, false discovery rate; HFHS, high-fat, high-sucrose. *n* = 3 mice/group.

**Table 4.** GSEA enrichment analysis (Biological Processes) in male cardiac tissues in response to 1-mo HFHS feeding

Name	NES	FDR q-Val
GOBP_LIPID_OXIDATION	2.1	0.027
GOBP_FATTY_ACID_CATABOLIC_PROCESS	2.0	0.030
GOBP_EXTERNAL_ENCAPSULATING_STRUCTURE_ORGANIZATION	2.0	0.032
GOBP_FATTY_ACID_BETA_OXIDATION	2.1	0.035
GOBP_REGULATION_OF_FATTY_ACID_OXIDATION	2.1	0.046
GOBP_RIBOSOME_BIOGENESIS	−2.5	0.000
GOBP_RRNA_METABOLIC_PROCESS	−2.4	0.000
GOBP_NCRNA_PROCESSING	−2.4	0.000
GOBP_RIBONUCLEOPROTEIN_COMPLEX_BIOGENESIS	−2.4	0.000
GOBP_TRNA_METABOLIC_PROCESS	−2.2	0.000
GOBP_TRNA_MODIFICATION	−2.2	0.000
GOBP_RIBOSOMAL_SMALL_SUBUNIT_BIOGENESIS	−2.2	0.000
GOBP_MATURATION_OF_SSU_RRNA	−2.2	0.000
GOBP_RIBOSOMAL_LARGE_SUBUNIT_BIOGENESIS	−2.1	0.000
GOBP_RNA_MODIFICATION	−2.1	0.001
GOBP_MATURATION_OF_5_8S_RRNA	−2.1	0.001
GOBP_TRNA_PROCESSING	−2.1	0.001
GOBP_MATURATION_OF_5_8S_RRNA_FROM_TRICISTRONIC_RRNA_TRANSCRIPT_SSU_RRNA_5_8S_RRNA_LSU_RRNA	−2.1	0.001
GOBP_CYTOPLASMIC_TRANSLATION	−2.1	0.002
GOBP_REGULATION_OF_RRNA_PROCESSING	−2.1	0.002
GOBP_RNA_PHOSPHODIESTER_BOND_HYDROLYSIS	−2.0	0.002
GOBP_TRANSLATION_AT_SYNAPSE	−2.0	0.002
GOBP_CLEAVAGE_INVOLVED_IN_RRNA_PROCESSING	−2.1	0.002
GOBP_RIBOSOME_ASSEMBLY	−2.0	0.003
GOBP_MATURATION_OF_SSU_RRNA_FROM_TRICISTRONIC_RRNA TRANSCRIPT_SSU_RRNA_5_8S_RRNA_LSU_RRNA	−2.0	0.003
GOBP_RESPONSE_TO_FUNGUS	−2.0	0.005
GOBP_NUCLEIC_ACID_PHOSPHODIESTER_BOND_HYDROLYSIS	−2.0	0.006
GOBP_RNA_PHOSPHODIESTER_BOND_HYDROLYSIS_ENDONUCLEOLYTIC	−1.9	0.009
GOBP_NEGATIVE_REGULATION_OF_DNA_RECOMBINATION	−1.9	0.018
GOBP_ENDONUCLEOLYTIC_CLEAVAGE_INVOLVED_IN_RRNA_PROCESSING	−1.9	0.018

FDR, false discovery rate; GSEA, gene set enrichment analysis; HFHS, high-fat, high-sucrose; NES, normalized enrichment score. *n* = 3 mice/group.

**Table 5.** GSEA enrichment analysis (Canonical Pathways) on male cardiac tissue in response to 1-mo HFHS feeding

Name	NES	FDR q-Val
REACTOME_MITOCHONDRIAL_FATTY_ACID_BETA_OXIDATION	2.2	0.000
WP_FATTY_ACID_BETAOXIDATION	2.1	0.005
WP_WHITE_FAT_CELL_DIFFERENTIATION	2.1	0.005
REACTOME_COLLAGEN_BIOSYNTHESIS_AND_MODIFYING_ENZYMES	2.0	0.007
REACTOME_SPHINGOLIPID_DE_NOVO_BIOSYNTHESIS	2.0	0.009
REACTOME_FATTY_ACID_METABOLISM	2.0	0.015
REACTOME_PKA_MEDIATED_PHOSPHORYLATION_OF_CREB	1.9	0.019
REACTOME_MET_ACTIVATES_PTK2_SIGNALING	1.9	0.018
REACTOME_SENSORY_PERCEPTION	1.9	0.022
WP_FATTY_ACID_BIOSYNTHESIS	1.9	0.022
WP_MITOCHONDRIAL_LONG_CHAIN_FATTY_ACID_BETAOXIDATION	1.9	0.023
REACTOME_METABOLISM_OF_LIPIDS	1.9	0.034
REACTOME_GLYCOSAMINOGLYCAN_METABOLISM	1.8	0.037
WP_GDNFRET_SIGNALING_AXIS	1.8	0.038
REACTOME_O_LINKED_GLYCOSYLATION_OF_MUCINS	1.8	0.036
REACTOME_CROSSLINKING_OF_COLLAGEN_FIBRILS	1.8	0.036
WP_SPHINGOLIPID_METABOLISM_OVERVIEW	1.8	0.037
REACTOME_ION_TRANSPORT_BY_P_TYPE_ATPASES	1.8	0.043
REACTOME_DARPP_32_EVENTS	1.8	0.045
REACTOME_O_LINKED_GLYCOSYLATION	1.8	0.044
REACTOME_RHO_GTPASES_ACTIVATE_PAKS	1.8	0.045
REACTOME_VISUAL_PHOTOTRANSDUCTION	1.8	0.044
REACTOME_MET_PROMOTES_CELL_MOTILITY	1.8	0.045
REACTOME_N_GLYCAN_ANTENNAE_ELONGATION_IN_THE_MEDIAL_ TRANS_GOLGI	1.8	0.047
REACTOME_COLLAGEN_FORMATION	1.8	0.046
REACTOME_MAJOR_PATHWAY_OF_RRNA_PROCESSING_IN_THE_NUCLEOLUS_AND_CYTOSOL	−2.4	0.000
WP_CYTOPLASMIC_RIBOSOMAL_PROTEINS	−2.2	0.000
REACTOME_EUKARYOTIC_TRANSLATION_INITIATION	−2.1	0.001
REACTOME_FORMATION_OF_A_POOL_OF_FREE_40S_SUBUNITS	−2.1	0.002
REACTOME_NONSENSE_MEDIATED_DECAY_NMD_INDEPENDENT_OF_ THE_EXON_JUNCTION_COMPLEX_EJC	−2.0	0.003
REACTOME_NONSENSE_MEDIATED_DECAY_NMD	−2.0	0.004
REACTOME_SNRNP_ASSEMBLY	−1.9	0.021
REACTOME_TRANSPORT_OF_MATURE_MRNAS_DERIVED_FROM_ INTRONLESS_TRANSCRIPTS	−1.9	0.028
REACTOME_ACTIVATION_OF_THE_MRNA_UPON_BINDING_OF_THE_CAP_BINDING_COMPLEX_AND_EIFS_AND_SUBSEQUENT_BINDING_TO_43S	−1.9	0.031
REACTOME_PROCESSING_OF_CAPPED_INTRON_CONTAINING_PRE_MRNA	−1.8	0.036
WP_TYPE_II_INTERFERON_SIGNALING_IFNG	−1.8	0.035
REACTOME_TRANSPORT_OF_THE_SLBP_DEPENDANT_MATURE_MRNA	−1.8	0.035
REACTOME_SUMOYLATION_OF_RNA_BINDING_PROTEINS	−1.8	0.038
REACTOME_PROCESSING_OF_DNA_DOUBLE_STRAND_BREAK_ENDS	−1.8	0.040
REACTOME_TRANSLATION	−1.8	0.043
REACTOME_G2_M_DNA_DAMAGE_CHECKPOINT	−1.8	0.043

FDR, false discovery rate; GSEA, gene set enrichment analysis; HFHS, high-fat, high-sucrose; NES, normalized enrichment score. *n* = 3 mice/group.

**Table 6.** ShinyGO Gene Ontology Biological Processes analysis showing up- and downregulated genes in female cardiac tissues in response to 1-mo HFHS diet

Pathway	Genes (*n*)	Pathway Genes (*n*)	Fold Enrichment	Enrichment FDR	Upregulated Genes (vs. Normal Chow Diet)	Downregulated Genes (vs. Normal Chow Diet)
Extracellular structure organization	62	288	6.1	7.99E-28	Loxl3 Col18a1 Col1a1 Col5a3 Fbln1 Sulf1 Myh11 Ccn2 Aebp1 Itgb3 Papln Cma1 Col14a1 Adamts20 Ccdc80 Vit Cyp1b1 Efemp2 Mmp19 Col5a2 Col3a1 Fn1 Dpt Itga8 Eng Col5a1 Slc2a10 Postn Sfrp2 Col15a1 Mmp23 Col1a2 Eln Col4a6 Comp Smpd3 Thsd4 Kif9 Antxr1 Adamts15 Rxfp1 Dpp4 Adamts2 Olfml2b Ltbp4 Col16a1 Sh3pxd2b Npnt Fmod Foxf1 Adamts6 Olfml2a Foxc2 Adamts12 Ndnf Adamts19 Slc39a8 Adamts14 Col23a1 Adamtsl1 Col4a4	Scx
External encapsulating structure organization	62	287	6.1	7.99E-28	Loxl3 Col18a1 Col1a1 Col5a3 Fbln1 Sulf1 Myh11 Ccn2 Aebp1 Itgb3 Papln Cma1 Col14a1 Adamts20 Ccdc80 Vit Cyp1b1 Efemp2 Mmp19 Col5a2 Col3a1 Fn1 Dpt Itga8 Eng Col5a1 Slc2a10 Postn Sfrp2 Col15a1 Mmp23 Col1a2 Eln Col4a6 Comp Smpd3 Thsd4 Kif9 Antxr1 Adamts15 Rxfp1 Dpp4 Adamts2 Olfml2b Ltbp4 Col16a1 Sh3pxd2b Npnt Fmod Foxf1 Adamts6 Olfml2a Foxc2 Adamts12 Ndnf Adamts19 Slc39a8 Adamts14 Col23a1 Adamtsl1 Col4a4	Scx
Extracellular matrix organization	62	286	6.1	7.99E-28	Loxl3 Col18a1 Col1a1 Col5a3 Fbln1 Sulf1 Myh11 Ccn2 Aebp1 Itgb3 Papln Cma1 Col14a1 Adamts20 Ccdc80 Vit Cyp1b1 Efemp2 Mmp19 Col5a2 Col3a1 Fn1 Dpt Itga8 Eng Col5a1 Slc2a10 Postn Sfrp2 Col15a1 Mmp 23 Col1a2 Eln Col4a6 Comp Smpd3 Thsd4 Kif9 Antxr1 Adamts15 Rxfp1 Dpp4 Adamts2 Olfml2b Ltbp4 Col16a1 Sh3pxd2b Npnt Fmod Foxf1 Adamts6 Olfml2a Foxc2 Adamts12 Ndnf Adamts19 Slc39a8 Adamts14 Col23a1 Adamtsl1 Col4a4	Scx
Cell adhesion	126	1,362	2.6	1.20E-20	Bmp10 Cdh3 Adipoq Msln Gata5 Pck1 Nfasc Plet1 Adam12 Slc39a8 Plekha7 Comp Mbp Spon2 Npnt Vsig4 Cd24a Col12a1 Foxf1 Cdhr4 Ephb2 Itgb4 Dchs2 Srcin1 Tnc Ccn2 Cldn15 Dpp4 Unc13d Megf11 Itgb3 Itga8 Ndnf Susd5 Gcnt1 Ccn3 Ephb6 Prkcz Fat3 Lsamp Gpnmb Col3a1 Bst1 Thbs1 Cyp1b1 Dusp26 Vit Sdk2 Hpse P2ry12 Foxc2 Adamts12 Col14a1 Plxnc1 Fn1 Plxna3 Col18a1 Postn Cldn34c1 Aoc3 Ncam1 Col1a1 Ubash3b Ttyh1 Ptk7 Dmtn Cdon Antxr1 Col5a3 Bcl6 Angpt2 Kif26b Sspo Clstn2 Adam19 Col15a1 Radil Flna Dbn1 Plaur Col6a3 Nfkbiz Col5a1 Sdk1 Fstl3 Sulf1 Dpt Fbln1 Can Efs Dlg2 Stab1 Smad6 Il27ra Sfrp1 Sorbs3 Eng Ccdc80 Zyx Fbn1 Spon1 Loxl3 Plxna4 Cadm3 Col6a2 Pcdhgc3 Gsn Efemp2 Thy1 Col16a1 Egfr Mcam Lrrc32 Svep1 Mxra8 Dchs1 Adam23 S txbp1	Ephb3 Lrrc4b Cadm4 Nat8f1 Bmp7 Myot Vinac1 Cdk5r1
Biological adhesion	126	1,374	2.6	2.09E-20	Bmp10 Cdh3 Adipoq Msln Gata5 Pck1 Nfasc Plet1 Adam12 Slc39a8 Plekha7 Comp Mbp Spon2 Npnt Vsig4 Cd24a Col12a1 Foxf1 Cdhr4 Ephb2 Itgb4 Dchs2 Srcin1 Tnc Ccn2 Cldn15 Dpp4 Unc13d Megf11 Itgb3 Itga8 Ndnf Susd5 Gcnt1 Ccn3 Ephb6 Prkcz Fat3 Lsamp Gpnmb Col3a1 Bst1 Thbs1 Cyp1b1 Dusp26 Vit Sdk2 Hpse P2ry12 Foxc2 Adamts12 Col14a1 Plxnc1 Fn1 Plxna3 Col18a1 Postn Cldn34c1 Aoc3 Ncam1 Col1a1 Ubash3b Ttyh1 Ptk7 Dmtn Cdon Antxr1 Col5a3 Bcl6 Angpt2 Kif26b Sspo Clstn2 A dam19 Col15a1 Radil Flna Dbn1 Plaur Col6a3 Nfkbiz Col5a1 Sdk1 Fstl3 Sulf1 Dpt Fbln1 Can Efs Dlg2 Stab1 Smad6 Il27ra Sfrp1 Sorbs3 Eng Ccdc80 Zyx Fbn1 Spon1 Loxl3 Plxna4 Cadm3 Col6a2 Pcdhgc3 Gsn Efemp2 Thy1 Col16a1 Egfr Mcam Lrrc32 Svep1 Mxra8 Dchs1 Ad am23 Stxbp1	Ephb3 Lrrc4b Cadm4 Nat8f1 Bmp7 Myot Vinac1 Cdk5r1
Animal organ morphogenesis	94	1,041	2.6	4.02E-14	Bmp10 Gata5 Fras1 Shank2 Slc24a4 Chad Ntrk2 Acp5 Vdr Npy1r Comp Tgfa Actg2 Npnt Cyp26b1 Wnt9b Rp1 Fst Atp2b2 Hoxa5 Efemp1 Mdfi Foxf1 Tmem119 Slit3 Ephb2 Itgb4 Gpc3 Olfm1 Tnc Smpd3 Ccn2 Igfbp5 Megf1 1 Acta2 Pam Itga8 Sfrp2 Duox2 Gcnt1 Adgrg6 Osr1 Fat3 Twist1 Gja5 Col3a1 Inhba Myc Sdk2 Nkd1 Fjx1 Pdgfc Foxc2 Sema3a Eln Col1a1 Gnao1 Ptk7 Cdon Kif26b Col1a2 Six1 Flna Col5a1 Sdk1 Sulf1 Tsku Mki67 Sh3pxd2b Insig1 Nbl1 Plekha4 Smad6 Sfrp1 Eng Col5a2 Fb n1 Msx1 Mfap5 Efemp2 Thy1 Egfr Dchs1	Agtr1a Tcap Scube2 Myl2 Irx3 Myl3 Scx Fgf18 Bmp7 Irx5 Irx1
Enzyme-linked receptor protein signaling pathway	86	907	2.7	4.68E-14	Bmp10 Cdh3 Fgf12 Adipoq Bmp3 Nppa Ntrk2 Chrdl1 Ntrk3 Sfrp5 Ahsg Comp Col4a6 Tgfa Sost Npnt Dok5 Fst Sh2b2 Efemp1 Ephb2 Slc2a10 Gpc3 Smpd3 Ccn2 Tmem108 Igfbp5 Itgb3 Itga8 Sfrp2 T xnip Ccn3 Ephb6 Prkcz Col3a1 Inhba Thbs1 Sort1 Igfbp6 Myof Shcbp1 Pdgfc Foxc2 Irs2 Adamts12 Creb3l1 Igfbp4 Col1a1 Ubash3b Angpt2 Col1a2 Hap1 Dok2 Cidea Adgra2 Plaur Fstl3 Sulf1 Tgfb1i1 Tsku Vim Ndn Fstl1 Nbl1 Efs Smad6 Sfrp1 Eng Zyx Fbn1 Igfbp3 Msx1 Plat Egfr Ldlrad4 Lrrc32 Svep1 Ltbp4	Ephb3 Cadm4 Scx Fgf18 Hspa5 Pdzd3 Bmp7 Cdk5r1
Response to endogenous stimulus	117	1,606	2.1	2.55E-11	Bmp10 Ucp1 Tat Slc26a3 Fgf12 Adipoq Rxfp1 Gata5 Krt19 Pck1 Nppa Ntrk2 Cdo1 Chrdl1 Ntrk3 Cib2 Sfrp5 Ahsg Comp Col4a6 Sost Atp2a1 Npnt Cacna1h S100b Fst Cd24a Sh2b2 Ptgfr Chrm3 Slit3 Slc2a10 Gpc 3 Tnc Brca1 Timp1 Cfb Smpd3 Ryr3 Ccn2 Tmem108 Igfbp5 Npas4 Pam Itgb3 Itga8 Ndnf Sfrp2 Txnip Pappa Gcnt1 Kcnc2 Prkcz Col3a1 Inhba Myc Thbs1 Dhh Sort1 Shcbp1 Pdgfc P2ry12 Foxc2 Irs2 Adamts12 Trem2 Ucp2 Atp1a3 Postn Creb3l1 Col1a1 Map1b Gnao1 Dmtn Aplp1 Col1a2 Six1 Ldlr Flna Cidea Klf10 Dbn1 Fstl3 Sulf1 Tgfb1i1 Tsku Vim Mmp19 Klf11 Insig1 Fstl1 Nbl1 Smad6 Sfrp1 Eng Col5a2 Zyx Fbn1 Serpinf1 Msx1 Col16a1 Egfr Ldlrad4 Eef1a1 Lrrc32 Ltbp4 Baiap2	Agtr1a Gpt Timp4 Shmt1 Ptprn Scx Fgf18 Hspa5 Bmp7 Htr7
Anatomical structure formation involved in morphogenesis	93	1,144	2.3	2.64E-11	Bmp10 Gata5 Krt19 Scgb3a1 Nfasc Krt8 Slc24a4 Chad Lrp8 Adam12 Angptl4 Tgfa Wnt9b Atp2b2 Hoxa5 Cma1 Col12a1 Foxf1 Ephb2 Itgb4 Ccl8 Ism1 Olfm1 C3 Brca1 Smpd3 Ccn2 Unc13d Megf11 Itgb3 Ndnf Sfrp2 Adgrg6 Osr1 Ccn3 Fat3 Twist1 Gja5 Inhba Thbs1 Csrp2 Cyp1b1 Sdk2 Hpse Myof Fjx1 Ptgis Foxc2 Trem2 Fn1 Col18a1 Creb3l1 Ecm1 Col1a1 Ptk7 Cdon Bcl6 Angpt2 Kif26b Six1 Col15a1 Flna Anpep Adgra2 Col5a1 Sdk1 Sulf1 Arhgap22 Mmp19 Sta b1 Sfrp1 Eng Adamts15 Col5a2 Meox1 Serpinf1 Msx1 Thy1 Mcam Csrp1 Dchs1	Agtr1a Ephb3 Tcap Myl2 Irx3 Scx Tmod4 Fgf18 Tnnt1 Bmp7 Irx1 Cdk5r1
Tissue development	131	1,900	2	2.91E-11	Bmp10 Cdh3 Adipoq Bmp3 Rxfp1 Gata5 Pck1 Fras1 Nppa Upk1b Plet1 Vdr Ntrk3 Ahsg Lgals3 Comp Actg2 Npnt Cyp26b1 Wnt9b S100b Fst Atp2b2 Cd24a Hoxa5 Efemp1 Tubb3 Col12a1 Foxf1 Tmem119 Irf6 Itgb4 Gpc3 Evpl Olfm1 C 3 Tnc Timp1 Lrrc17 Smpd3 Ccn2 Cytl1 Igfbp5 Acta2 Tppp3 Itgb3 Itga8 Sfrp2 Duox2 Txnip Gcnt1 Sema3d Osr1 Ccn3 Tagln Creb3l2 Twist1 Gja5 Gpnmb Col3a1 Inhba Myc Slc24a3 Csrp2 Cyp1b1 Iqgap3 Hpse Nkd1 Ptgis Foxc2 Sema3a Adamts12 Col14a1 Fn1 Sgms2 Nupr1 Col 18a1 Postn Ecm1 Eln Col1a1 Ptk7 Rcn3 Cdon Angpt2 Kif26b Col1a2 Six1 Col15a1 Radil Flna Klf10 Plaur Col5a1 Sulf1 Tgfb1i1 Vim Sh3pxd2b Fstl1 Plekha4 Evc Smad6 Sfrp1 Eng Col5a2 Arhgef26 Maff Loxl3 Meox1 Msx1 Efemp2 Egfr Ldlrad4 Csrp1 Svep1 Dchs1	Agtr1a Tcap Scube2 Myl2 Irx3 Kcne1 Dnase1l2 Myl3 Scx Gsta3 Sema5b Fgf18 Mylpf Bmp7 Irx1
Collagen fibril organization	18	53	9.6	6.75E-11	Fmod Comp Aebp1 Sfrp2 Adamts14 Col3a1 Cyp1b1 Foxc2 Col14a1 Col1a1 Col1a2 Adamts2 Col5a1 Dpt Col5a2 Loxl3 Efemp2	Scx
Response to growth factor	66	694	2.7	1.21E-10	Bmp10 Fgf12 Gata5 Lrp8 Ntrk2 Chrdl1 Ntrk3 Sfrp5 Comp Sost Npnt Dok5 Fst Slc2a10 Gpc3 Tnc Smpd3 Ccn2 Tmem108 Itgb3 Itga8 Ndnf Sfrp2 Kcnc2 Twist1 Col3a1 Myc Thbs1 Sort1 Myof Shcbp1 Adamts12 Postn Creb3l 1 Col1a1 Map1b Angpt2 Col1a2 Hap1 Cidea Adgra2 Dbn1 Fstl3 Sulf1 Tgfb1i1 Tsku Ndn Fstl1 Nbl1 Smad6 Sfrp1 Eng Zyx Fbn1 Msx1 Egfr Ldlrad4 Eef1a1 Lrrc32 Ltbp4 Baiap2	Cadm4 Scx Fgf18 Hspa5 Bmp7
Supramolecular fiber organization	68	728	2.7	1.21E-10	Bmp10 Krt19 Krt8 Fmod Comp Aif1l Tpm2 Rp1 Hspa1b Fhdc1 Aebp1 Ccn2 Ltbp2 Hspa1a Tppp3 Diaph3 Sfrp2 Elmo3 Pdlim3 Adamts14 Col3a1 Cyria Csrp2 Cyp1b1 Lmod1 Foxc2 Col14a1 Eln Col1a1 Map1b Dmtn Cgnl 1 Col1a2 Ldlr Flna Marcksl1 Adamts2 Dbn1 Myh11 Thsd4 Col5a1 Vim Tmsb10 Dpt Sh3pxd2b Map1a Efs Sorbs3 Col5a2 Zyx Loxl3 Tubb4a Gsn Mfap5 Capg Efemp2 Csrp1 Ltbp4 Arrb1 Baiap2 Clip2	Tcap Fign Myl2 Scx Tmod4 Mid1ip1 Tnnt1
Cellular response to growth factor stimulus	64	666	2.7	1.66E-10	Bmp10 Fgf12 Gata5 Lrp8 Ntrk2 Chrdl1 Ntrk3 Sfrp5 Comp Sost Npnt Dok5 Fst Slc2a10 Gpc3 Smpd3 Ccn2 Tmem108 Itgb3 Itga8 Ndnf Sfrp2 Twist1 Col3a1 Myc Thbs1 Sort1 Myof Shcbp1 Adamts12 Post n Creb3l1 Col1a1 Map1b Angpt2 Col1a2 Hap1 Cidea Adgra2 Dbn1 Fstl3 Sulf1 Tgfb1i1 Tsku Ndn Fstl1 Nbl1 Smad6 Sfrp1 Eng Zyx Fbn1 Msx1 Egfr Ldlrad4 Eef1a1 Lrrc32 Ltbp4 Baiap2	Cadm4 Scx Fgf18 Hspa5 Bmp7
Cell-cell signaling	107	1,496	2	6.43E-10	Cdh3 Fgf12 Adipoq Dkk3 Nppa Shank2 Nnat Lypd1 Lrp8 Ecrg4 Rab3b Ntrk2 Prkar2b Dio2 Nptx1 Cacna2d2 Npy1r Sfrp5 Adcy1 Rspo1 Dlgap1 Rab11fip1 Sost Wnt9b Ildr2 S100b Atp2b2 Cd24a Dkk2 Slc24a2 Hoxa5 Cacna1d Chrm3 Slc18a2 Mdfi Ephb2 Gpc3 Slc17a7 C3 Tnc Grik2 Panx2 Smpd3 Tmem108 Npas4 Chrna2 Nphp4 Sfrp2 Gabre Ccn3 Daam2 Prkcz Ppfia2 Gja5 Gpnmb Inhba Myc Dhh Shisa3 Nkd1 Fjx1 P2ry12 Cadps2 Irs2 Sv2b Gabra3 Ucp2 Gipr Col1a1 Map1b Ptk7 Clstn2 Hap1 Flna Adgra2 Dbn1 Sulf1 Tgfb1i1 Tsku Rin1 Map1a Unc13b Dtnb Dlg2 Plekha4 Stab1 Sfrp1 Slc16a2 Igfbp3 Plat Thy1 Egfr F2r Ltbp4 Arrb1 Baiap2 Stxbp1	Gpr27 Agtr1a Ptprn Rasd2 Nr0b2 Lgi1 Htr7 Nrn1 Cdk5r1 Apba2
Cellular response to endogenous stimulus	98	1,323	2.1	7.95E-10	Bmp10 Ucp1 Slc26a3 Fgf12 Adipoq Rxfp1 Gata5 Pck1 Ntrk2 Chrdl1 Ntrk3 Cib2 Sfrp5 Ahsg Comp Col4a6 Sost Npnt Cacna1h Fst Sh2b2 Ptgfr Chrm3 Slit3 Slc2a10 Gpc3 Tnc Brca1 Smpd3 Ryr3 Ccn2 Tme m108 Igfbp5 Npas4 Itgb3 Itga8 Ndnf Sfrp2 Prkcz Col3a1 Inhba Myc Thbs1 Sort1 Shcbp1 Pdgfc P2ry12 Foxc2 Irs2 Adamts12 Trem2 Ucp2 Atp1a3 Postn Creb3l1 Col1a1 Map1b Gnao1 Dmtn Aplp1 Col1a2 Six1 Flna Cidea Klf10 Dbn1 Fstl3 Sulf1 Tgfb1i1 Tsku Vim Klf11 Ins ig1 Fstl1 Nbl1 Smad6 Sfrp1 Eng Col5a2 Zyx Fbn1 Serpinf1 Msx1 Col16a1 Egfr Ldlrad4 Eef1a1 Lrrc32 Ltbp4 Baiap2	Agtr1a Gpt Shmt1 Scx Fgf18 Hspa5 Bmp7 Htr7
Circulatory system development	88	1,135	2.2	1.00E-09	Bmp10 Gata5 Nppa Ntrk2 Adam12 Ntrk3 Npy1r Comp Angptl4 Tgfa Hoxa5 Cma1 Foxf1 Slit3 Ephb2 Slc2a10 Gpc3 Ism1 Olfm1 C3 Brca1 Ccn2 Acta2 Pam Itgb3 Ndnf Sfrp2 Adgrg6 Osr1 Ccn3 Pdlim3 Twist1 Gja5 Col3 a1 Thbs1 Cyp1b1 Hpse Ptgis Foxc2 Col14a1 Fn1 Col18a1 Creb3l1 Ecm1 Eln Col1a1 Ptk7 Antxr1 Angpt2 Adam19 Col1a2 Six1 Col15a1 Ldlr Flna Anpep Adgra2 Col5a1 Sulf1 Arhgap22 Mmp19 Sh3pxd2b Loxl1 Stab1 Smad6 Eng Fbn1 Ece1 Adamts6 Serpinf1 Msx1 Efemp2 Thy1 E gfr Mcam Pdlim2 Svep1 Dchs1	Agtr1a Ephb3 Tcap Myl2 Irx3 Myl3 Scx Fgf18 Bmp7 Cth
Cell migration	104	1,474	2	2.62E-09	Bmp10 Adipoq Plet1 Lrp8 Ntrk2 Ntrk3 Lgals3 Gpm6a Stk26 Camk1d Cd24a Hoxa5 Efemp1 Foxf1 Ephb2 Itgb4 Ccl8 Gpc3 Plvap Olfm1 Srcin1 Timp1 Smpd3 Ccn2 Dpp4 Igfbp5 Acta2 Itgb3 Ndnf Sfrp2 Gcnt1 Sema3d Ccn3 Elmo3 Prkcz F at3 Twist1 Gpnmb Col3a1 Myc Bst1 Thbs1 Cyp1b1 Prr5l Dclk1 Igfbp6 Pdgfc P2ry12 Foxc2 Sema3a Irs2 Adamts12 Anln Trem2 Plxnc1 Fn1 Plxna3 Col18a1 Postn Aoc3 Ecm1 Col1a1 Ptk7 Dmtn Angpt2 Six1 Trp53inp1 Radil Flna Adgra2 Col5a1 Sulf1 Vim Tmsb10 Ndn Cd248 F stl1 Fbln1 Nbl1 Efs Il27ra Sfrp1 Eng S100a11 Plxna4 Igfbp3 Serpinf1 Plat Thy1 Egfr Ldlrad4 Tns3 F2r Mcam Svep1 Dchs1	Agtr1a Ephb3 Sema5b Fgf18 Hspa5 Bmp7 Vinac1 Cdk5r1
Locomotion	119	1,822	1.9	8.87E-09	Bmp10 Adipoq Nfasc Plet1 Lrp8 Ntrk2 Dnah6 Ntrk3 Lgals3 Dpysl5 Gpm6a Stk26 Camk1d Atp2b2 Cd24a Hoxa5 Efemp1 Tubb3 Foxf1 Slit3 Ephb2 Itgb4 Ccl8 Gpc3 Plvap Olfm1 Srcin1 Timp1 Smpd3 Ccn2 Adcy3 Dpp4 Igfbp5 Acta2 Nphp4 Itgb3 Ndnf Sfrp2 Duox2 Gcnt1 Sema3d Ccn3 Elmo3 Ephb6 Prkcz Fat3 Twist1 Gpnmb Col3a1 Myc Bst1 Thbs1 Cyp1b1 Prr5l Dclk1 Igfbp6 Pdgfc P2ry12 Foxc2 Sema3a Irs2 Adamts12 Anln Trem2 Plxnc1 Fn1 Plxna3 Col18a1 Postn Aoc3 Ecm1 Col1a1 Ptk7 Dmtn Angpt2 Six1 Trp 53inp1 Radil Flna Adgra2 Dbn1 Col5a1 Etv1 Sulf1 Vim Tmsb10 Ndn Cd248 Fstl1 Fbln1 Nbl1 Efs Il27ra Sfrp1 Eng S100a11 Plxna4 Igfbp3 Serpinf1 Plat Thy1 Egfr Ldlrad4 Tns3 F2r Mcam Svep1 Dchs1	Agtr1a Ephb3 Cadm4 Sema5b Fgf18 Hspa5 Bmp7 Lgi1 Myot Vinac1 Cdk 5r1
Regulation of cell differentiation	109	1,610	1.9	8.87E-09	Bmp10 Bnc1 Adipoq Gata5 Pck1 Olfm2 Lrp8 Ntrk2 Vdr Ntrk3 Adig Npnt Cyp26b1 Wnt9b S100b Fst Cd24a Hoxa5 Efemp1 Tmem119 Ephb2 Ccdc3 Ccl8 Smoc1 Ankle1 Olfm1 Lrrc17 Nmrk2 Ccn2 Igfbp5 Unc13d Itgb 3 Sfrp2 Sema3d Osr1 Ccn3 Daam2 Prkcz Aspa Twist1 Inhba Myc Sort1 Adamts20 Rbp1 Zfp365 P2ry12 Zbtb7c Sema3a Tiam2 Adamts12 Trem2 Plxnc1 Fn1 Plxna3 Postn Col1a1 Map1b Ubash3b Dmtn Cdon Slc45a3 Bcl6 Six1 Hap1 Trp53inp1 Ldlr Flna Klf10 Adgra2 Dbn1 Nfkbiz Col5a1 Fstl3 Tgfb1i1 Tsku Vim Sh3pxd2b Insig1 Fbln1 Nbl1 Smad6 Sfrp1 Eng Islr2 Col5a2 Fbn1 Timp2 Maff Loxl3 Plxna4 Igfbp3 Serpinf1 Msx1 Rgs6 Efemp2 Thy1 Egfr Ldlrad4 Baiap2	Scube2 Irx3 Sema5b Fgf18 Bmp7 Gnb3 Cth Cdk5r1 Helt

Genes (*n*) refers to number of differentially expressed genes in the specified pathway. Pathway genes (*n*) refers to the total number of genes in the specified pathway. FDR, false discovery rate. HFHS, high-fat, high-sucrose. *n* = 3 mice/group.

**Table 7.** ShinyGO KEGG pathway analysis showing up- and downregulated genes in female cardiac tissues in response to 1-mo HFHS feeding

Pathway	Genes (*n*)	Pathway Genes (*n*)	Fold Enrichment	Enrichment FDR	Upregulated Genes (vs. Normal Chow Diet)	Downregulated Genes (vs. Normal Chow Diet)
Protein digestion and absorption	23	108	6	8.43E-10	Slc7a7 Col18a1 Col1a1 Col5a3 Col6a2 Slc7a8 Col14a1 Kcnk5 Col5a2 Col3a1 Col5a1 Col15a1 Col1a2 Eln Col4a6 Col12a1 Dpp4 Xpnpep2 Col16a1 Atp1a3 Col6a3 Col23a1 Col4a4	
ECM-receptor interaction	16	88	5.2	9.26E-06	Col1a1 Col6a2 Itgb3 Itgb4 Fn1 Itga8 Tnc Col1a2 Comp Fras1 Chad Thbs1 Npnt Col6a3 Sv2b Col4a4	
Focal adhesion	21	200	3	8.08E-04	Col1a1 Egfr Col6a2 Myl7 Itgb3 Itgb4 Fn1 Itga8 Pdgfc Tnc Col1a2 Zyx Col4a6 Flna Comp Chad Thbs1 Col6a3 Col4a4	Myl2 Mylpf
Relaxin signaling pathway	16	129	3.5	8.89E-04	Col1a1 Arrb1 Egfr Adcy1 Adcy3 Col3a1 Creb3l1 Prkcz Col1a2 Gnao1 Rxfp1 Acta2 Creb3l2 Gng2 Col4a4	Gnb3
Adrenergic signaling in cardiomyocyte	17	149	3.2	1.17E-03	Cacna2d2 Cacna1d Adcy1 Adcy3 Atp2a3 Cacna2d3 Creb3l1 Tpm2 Atp2b2 Atp2a1 Creb3l2 Atp1a3 Myl4	Myl2 Kcne1 Agtr1a Myl3
Dilated cardiomyopathy	13	94	3.9	1.19E-03	Cacna2d2 Cacna1d Adcy1 Adcy3 Itgb3 Itgb4 Atp2a3 Cacna2d3 Itga8 Tpm2 Atp2a1	Myl2 Myl3
Glycine, serine and threonine metabolism	8	40	5.7	2.58E-03	Chdh Aoc3 Psat1 Sds	Gnmt Gcat Shmt1 Cth
PI3K-Akt signaling pathway	28	357	2.2	2.58E-03	Col1a1 Brca1 Egfr Col6a2 Itgb3 Itgb4 Myc Fn1 Itga8 Creb3l1 Pck1 Pdgfc Tnc Col1a2 Tgfa Col4a6 Angpt2 Comp Creb3l2 Chad Thbs1 Gng2 Col6a3 F2r Ntrk2 Fgf18 Col4a4	Gnb3
Hypertrophic cardiomyopathy	12	91	3.7	2.58E-03	Cacna2d2 Cacna1d Itgb3 Itgb4 Atp2a3 Cacna2d3 Itga8 Tpm2 Prkag2 Atp2a1	Myl2 Myl3
Apelin signaling pathway	15	136	3.1	2.60E-03	Ccn2 Adcy1 Adcy3 Prkag2 Plin1 Plat Ucp1 Acta2 Gng2 Ryr3 Myl4	Myl2 Gnb3 Myl3 Agtr1a
Regulation of actin cytoskeleton	19	219	2.5	7.49E-03	Myh11 Egfr Myl7 Itgb3 Itgb4 Spata13 Diaph3 Baiap2 Fn1 Itga8 Gsn Pdgfc Iqgap3 Chrm3 F2r	Myl2 Mylpf Actr3b Fgf18
Complement and coagulation cascades	11	91	3.4	8.24E-03	C3 C2 A2m Plat Vsig4 Plaur F2r C1ra Cfd C4b Cfb	
Aldosterone synthesis and secretion	11	102	3.1	1.80E-02	Cacna1d Adcy1 Adcy3 Cacna1h Creb3l1 Atp2b2 Ldlr Creb3l2 Atp1a3 Nppa	Agtr1a
Calcium signaling pathway	19	240	2.2	1.80E-02	Cacna1d Egfr Adcy1 Adcy3 Atp2a3 Pde1b Cacna1h Pdgfc Ptgfr Atp2b2 Atp2a1 Chrm3 F2r Ntrk2 Ryr3 Ntrk3	Htr7 Agtr1a Fgf18
Cardiac muscle contraction	10	87	3.3	1.80E-02	Cacna2d2 Cacna1d Atp2a3 Cacna2d3 Tpm2 Atp2a1 Atp1a3 Myl4	Myl2 Myl3
Biosynthesis of unsaturated fatty acids	6	34	5	1.90E-02	Acaa1b Elovl7 Fads2 Scd1 Elovl6 Acot4	
PPAR signaling pathway	10	89	3.2	1.90E-02	Angptl4 Cpt1c Acaa1b Adipoq Fads2 Pck1 Hmgcs2 Plin1 Ucp1 Scd1	
TGF-β signaling pathway	10	95	3	2.95E-02	Fst Myc Fbn1 Thsd4 Smad6 Thbs1 Nbl1 Inhba Fmod	Bmp7
Glutamatergic synapse	11	113	2.8	3.18E-02	Gria3 Dlgap1 Cacna1d Adcy1 Adcy3 Gnao1 Shank2 Gng2 Grik2 Slc17a7	Gnb3
Circadian entrainment	10	98	3	3.35E-02	Gria3 Cacna1d Adcy1 Adcy3 Cacna1h Gnao1 Nos1ap Gng2 Ryr3	Gnb3

Genes (*n*) refers to number of differentially expressed genes in the specified pathway. Pathway genes (*n*) refers to the total number of genes in the specified pathway. FDR, false discovery rate; HFHS, high-fat, high-sucrose. *n* = 3 mice/group.

**Table 8.** GSEA enrichment analysis (Biological Processes) on female cardiac tissues in response to 1-mo HFHS feeding

Name	NES	FDR q-Val
GOBP_EXTERNAL_ENCAPSULATING_STRUCTURE_ORGANIZATION	2.4	0.000
GOBP_COLLAGEN_FIBRIL_ORGANIZATION	2.3	0.000
GOBP_POSITIVE_REGULATION_OF_SMOOTH_MUSCLE_CELL_MIGRATION	2.1	0.000
GOBP_EXTRACELLULAR_MATRIX_ASSEMBLY	2.1	0.001
GOBP_COLLAGEN_METABOLIC_PROCESS	2.1	0.000
GOBP_REGULATION_OF_EXTRACELLULAR_MATRIX_ORGANIZATION	2.0	0.001
GOBP_EXTRACELLULAR_MATRIX_DISASSEMBLY	2.0	0.001
GOBP_COLLAGEN_BIOSYNTHETIC_PROCESS	2.0	0.001
GOBP_PEPTIDE_CROSS_LINKING	2.0	0.001
GOBP_POSITIVE_REGULATION_OF_STEROID_METABOLIC_PROCESS	2.0	0.001
GOBP_APOPTOTIC_CELL_CLEARANCE	2.0	0.002
GOBP_PROTEOGLYCAN_METABOLIC_PROCESS	2.0	0.001
GOBP_CELL_SUBSTRATE_ADHESION	2.0	0.001
GOBP_POSITIVE_REGULATION_OF_CELL_SUBSTRATE_ADHESION	2.0	0.001
GOBP_TISSUE_REMODELING	2.0	0.002
GOBP_CELL_MATRIX_ADHESION	2.0	0.002
GOBP_REGULATION_OF_CELL_SUBSTRATE_ADHESION	2.0	0.002
GOBP_INTEGRIN_MEDIATED_SIGNALING_PATHWAY	2.0	0.002
GOBP_ADENYLATE_CYCLASE_MODULATING_G_PROTEIN_COUPLED_RECEPTOR_SIGNALING_PATHWAY	2.0	0.003
GOBP_MUCOPOLYSACCHARIDE_METABOLIC_PROCESS	2.0	0.003
GOBP_MIDDLE_EAR_MORPHOGENESIS	2.0	0.003
GOBP_POSITIVE_REGULATION_OF_FIBROBLAST_PROLIFERATION	1.9	0.003
GOBP_AMINOGLYCAN_METABOLIC_PROCESS	1.9	0.003
GOBP_SMOOTH_MUSCLE_CELL_MIGRATION	1.9	0.003
GOBP_MORPHOGENESIS_OF_A_POLARIZED_EPITHELIUM	1.9	0.003
GOBP_PROTON_MOTIVE_FORCE_DRIVEN_ATP_SYNTHESIS	−3.4	0
GOBP_MITOCHONDRIAL_RESPIRATORY_CHAIN_COMPLEX_ASSEMBLY	−3.4	0
GOBP_MITOCHONDRIAL_TRANSLATION	−3.4	0
GOBP_MITOCHONDRIAL_GENE_EXPRESSION	−3.3	0
GOBP_NADH_DEHYDROGENASE_COMPLEX_ASSEMBLY	−3.2	0
GOBP_OXIDATIVE_PHOSPHORYLATION	−3.2	0
GOBP_ATP_SYNTHESIS_COUPLED_ELECTRON_TRANSPORT	−3.1	0
GOBP_AEROBIC_RESPIRATION	−3.1	0
GOBP_AEROBIC_ELECTRON_TRANSPORT_CHAIN	−3.1	0
GOBP_CELLULAR_RESPIRATION	−2.9	0
GOBP_MITOCHONDRIAL_ELECTRON_TRANSPORT_NADH_TO_UBIQUINO	−2.7	0
GOBP_ATP_BIOSYNTHETIC_PROCESS	−2.6	0
GOBP_TRICARBOXYLIC_ACID_CYCLE	−2.6	0
GOBP_CYTOCHROME_COMPLEX_ASSEMBLY	−2.6	0
GOBP_ELECTRON_TRANSPORT_CHAIN	−2.6	0
GOBP_NUCLEOSIDE_TRIPHOSPHATE_BIOSYNTHETIC_PROCESS	−2.5	0
GOBP_ENERGY_DERIVATION_BY_OXIDATION_OF_ORGANIC_COMPOUND	−2.5	0
GOBP_PROTEIN_TARGETING_TO_MITOCHONDRION	−2.4	0
GOBP_PROTEIN_TRANSMEMBRANE_IMPORT_INTO_INTRACELLULAR_ORGANELLE	−2.4	0
GOBP_PROTEIN_LOCALIZATION_TO_MITOCHONDRION	−2.4	0
GOBP_PROTEIN_IMPORT_INTO_MITOCHONDRIAL_MATRIX	−2.4	0
GOBP_RESPIRATORY_CHAIN_COMPLEX_IV_ASSEMBLY	−2.3	0
GOBP_REGULATION_OF_MITOCHONDRIAL_GENE_EXPRESSION	−2.3	0
GOBP_REGULATION_OF_MITOCHONDRIAL_TRANSLATION	−2.3	0
GOBP_MITOCHONDRIAL_RNA_METABOLIC_PROCESS	−2.3	0

FDR, false discovery rate; GSEA, Gene set enrichment analysis; HFHS, high-fat, high-sucrose; NES, normalized enrichment score. *n* = 3 mice/group.

**Table 9.** GSEA enrichment analysis (Canonical Pathways) on female cardiac tissues in response to 1-mo HFHS feeding

Name	NES	FDR q-Val
REACTOME_ASSEMBLY_OF_COLLAGEN_FIBRILS_AND_OTHER_MULTIMERIC_STRUCTURES	2.4	0
REACTOME_EXTRACELLULAR_MATRIX_ORGANIZATION	2.2	0
REACTOME_INTEGRIN_CELL_SURFACE_INTERACTIONS	2.2	0
REACTOME_COLLAGEN_CHAIN_TRIMERIZATION	2.2	0
REACTOME_NON_INTEGRIN_MEMBRANE_ECM_INTERACTIONS	2.1	0
REACTOME_COLLAGEN_FORMATION	2.1	0
REACTOME_ELASTIC_FIBER_FORMATION	2.1	0
REACTOME_ECM_PROTEOGLYCANS	2.1	0
REACTOME_COLLAGEN_BIOSYNTHESIS_AND_MODIFYING_ENZYMES	2.1	0
WP_PROSTAGLANDIN_SYNTHESIS_AND_REGULATION	2.1	0
REACTOME_CROSSLINKING_OF_COLLAGEN_FIBRILS	2.0	0
REACTOME_MET_PROMOTES_CELL_MOTILITY	2.0	0
REACTOME_COLLAGEN_DEGRADATION	2.0	0
REACTOME_RESPONSE_TO_ELEVATED_PLATELET_CYTOSOLIC_CA2	2.0	0
REACTOME_MET_ACTIVATES_PTK2_SIGNALING	2.0	0
REACTOME_DEGRADATION_OF_THE_EXTRACELLULAR_MATRIX	2.0	0.001
WP_PRIMARY_FOCAL_SEGMENTAL_GLOMERULOSCLEROSIS_FSGS	1.9	0.001
REACTOME_MOLECULES_ASSOCIATED_WITH_ELASTIC_FIBRES	1.9	0.002
REACTOME_REGULATION_OF_INSULIN_LIKE_GROWTH_FACTOR_IGF_TRANSPORT_AND_UPTAKE_BY_INSULIN_LIKE_GROWTH_FACTOR_BINDING_PROTEINS_IGFBPS	1.9	0.004
WP_TYROBP_CAUSAL_NETWORK_IN_MICROGLIA	1.9	0.004
REACTOME_HEMOSTASIS	1.9	0.004
REACTOME_PLATELET_ACTIVATION_SIGNALING_AND_AGGREGATION	1.9	0.004
REACTOME_O_GLYCOSYLATION_OF_TSR_DOMAIN_CONTAINING_PROTEINS	1.8	0.006
REACTOME_GLYCOSAMINOGLYCAN_METABOLISM	1.8	0.008
WP_PPAR_SIGNALING_PATHWAY	1.8	0.009
REACTOME_RESPIRATORY_ELECTRON_TRANSPORT	−3.5	0
REACTOME_MITOCHONDRIAL_TRANSLATION	−3.3	0
REACTOME_RESPIRATORY_ELECTRON_TRANSPORT_ATP_SYNTHESIS_BY_CHEMIOSMOTIC_COUPLING_AND_HEAT_PRODUCTION_BY_UNCOUPLING_PROTEINS	−3.3	0
REACTOME_THE_CITRIC_ACID_TCA_CYCLE_AND_RESPIRATORY_ELECTRON_TRANSPORT	−3.2	0
REACTOME_COMPLEX_I_BIOGENESIS	−3.1	0
WP_ELECTRON_TRANSPORT_CHAIN	−3.0	0
WP_OXIDATIVE_PHOSPHORYLATION	−3.0	0
REACTOME_TRANSLATION	−2.8	0
REACTOME_MITOCHONDRIAL_BIOGENESIS	−2.7	0
REACTOME_UBIQUITIN_MEDIATED_DEGRADATION_OF_PHOSPHORYLATED_CDC25A	−2.6	0
REACTOME_DEGRADATION_OF_DVL	−2.5	0
REACTOME_DEGRADATION_OF_AXIN	−2.5	0
REACTOME_CDK_MEDIATED_PHOSPHORYLATION_AND_REMOVAL_OF_CDC6	−2.5	0
REACTOME_NUCLEAR_EVENTS_MEDIATED_BY_NFE2L2	−2.5	0
REACTOME_GLI3_IS_PROCESSED_TO_GLI3R_BY_THE_PROTEASOME	−2.5	0
REACTOME_CITRIC_ACID_CYCLE_TCA_CYCLE	−2.4	0
REACTOME_FORMATION_OF_ATP_BY_CHEMIOSMOTIC_COUPLING	−2.4	0
REACTOME_METABOLISM_OF_POLYAMINES	−2.4	0
WP_PROTEASOME_DEGRADATION	−2.4	0
REACTOME_STABILIZATION_OF_P53	−2.4	0
REACTOME_BRANCHED_CHAIN_AMINO_ACID_CATABOLISM	−2.4	0
REACTOME_HEDGEHOG_LIGAND_BIOGENESIS	−2.4	0
REACTOME_CROSS_PRESENTATION_OF_SOLUBLE_EXOGENOUS_ANTIGENS_ENDOSOMES	−2.4	0
REACTOME_REGULATION_OF_PTEN_STABILITY_AND_ACTIVITY	−2.4	0
REACTOME_CELLULAR_RESPONSE_TO_HYPOXIA	−2.4	0

FDR, false discovery rate; GSEA, Gene set enrichment analysis; HFHS, high-fat, high-sucrose; NES, normalized enrichment score. *n* = 3 mice/group.

**Figure 7.**
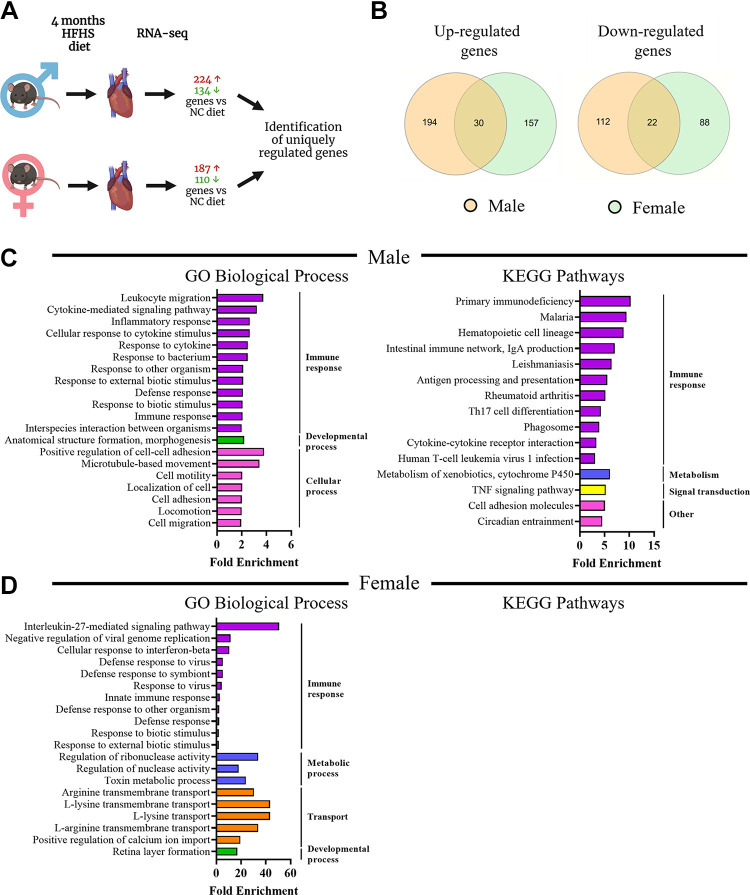
RNA-sequencing analysis of cardiac transcriptome in male and female mice after 4-mo of high-fat/high-sucrose diet. *A*: overview of RNA-seq analysis in male and female mouse hearts. Generated using Biorender.com. *B*: Venn diagram showing commonly and uniquely up- and downregulated genes in male and female mice in response to 4-mo high-fat/high-sucrose diet. *C*: gene enrichment analysis in genes uniquely up- and downregulated in male mice in response to high-fat/high-sucrose diet. *D*: gene enrichment analysis in genes uniquely up- and downregulated in female mice in response to high-fat/high-sucrose diet. For each analysis, the top 20 results that scored false discovery rate (FDR) <0.05 are displayed. *n* = 3 mice/group.

**Figure 8.**
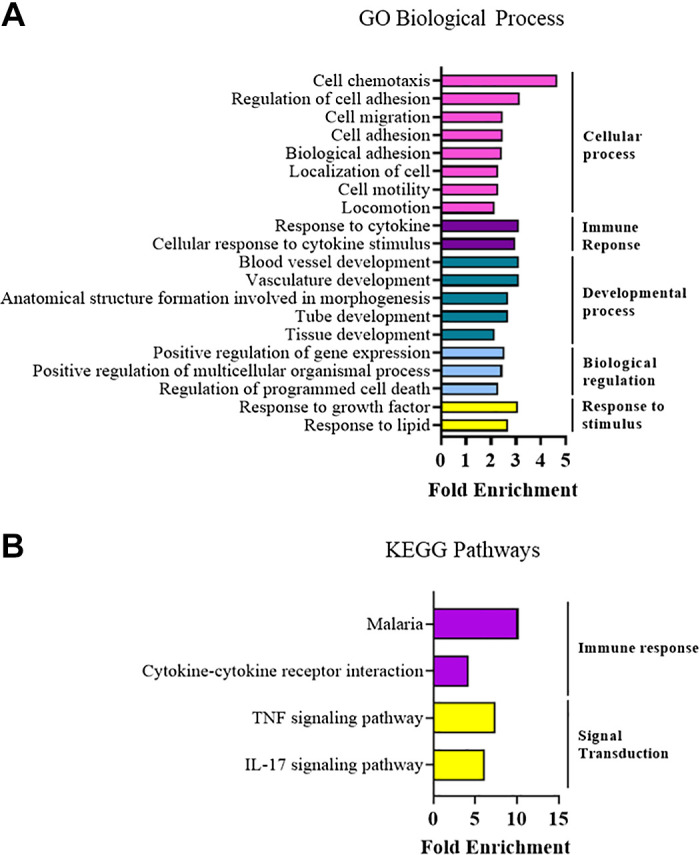
Enrichment analysis on differentially expressed genes between male and female mice in the absence of high-fat/high-sucrose (HFHS) diet at 4-mo time point. *A*: Gene ontology, biological processes. *B*: KEGG pathway analysis. For each analysis, the top 20 results that scored false discovery rate (FDR) <0.05 are displayed. *n* = 3 mice/group.

After 4 mo of diet, the cardiac gene expression response in females was markedly reduced (297 differentially expressed genes) compared with that observed at 1 mo of feeding. Males showed differential expression of 358 genes after 4 mo feeding (Fig. 7*A*). Similarly to 1 mo of feeding, we observed that most differentially expressed genes were unique to males or females. Comparison of the gene lists (Fig. 7*B*) showed that male mice had 194/224 genes uniquely upregulated and 110/134 genes uniquely downregulated, whereas females had 155/187 genes uniquely upregulated and 88/110 genes uniquely downregulated. ShinyGO enrichment analysis using GO Biological Processes and KEGG pathway databases was performed on uniquely regulated genes, which showed a number of immune system-related processes and pathways were prominent in male mice (Fig. 7, *C* and *D*, Tables 10 and 11), which were also featured in the GSEA analyses (Tables 12 and 13). Immune response-related pathways were also featured in female hearts after 4 mo of HFHS diet, although it should be noted that only 2 of 20 pathways were common between males and females (Table 14). Analysis of KEGG pathways in female mice did not yield any results that passed the false-discovery rate cut-off of 0.05, however, GSEA analysis showed enrichment of immune response, remodeling, and extracellular matrix-related Biological Processes and Canonical Pathways (Table 15 and 16). Analysis of male versus female cardiac gene expression without the influence of HFHS diet showed 110 upregulated and 189 downregulated genes in female mice compared with males at this time point. We found that there was enrichment of immune and developmental GO Biological Processes among the differentially expressed genes (Fig. 8*A*). KEGG pathway analysis highlighted immune pathways as well as TNF and IL-17 signaling pathways (Fig. 8*B*).

**Table 10.** ShinyGO Gene ontology Biological Processes analysis showing up- and downregulated genes in male cardiac tissues in response to 4-mo HFHS feeding

Pathway	Genes (*n*)	Pathway Genes (*n*)	Fold Enrichment	Enrichment FDR	Upregulated Genes (vs. Normal Chow Diet)	Downregulated Genes (vs. Normal Chow Diet)
Locomotion	49	1,822	2	2.08E-03	Dnah6 Cd24a Rsph9 Adam8 Itgax Ifitm1 Itgb7 Rgn Wdr66 Gli1 Ddx4 Ptpn22 Ccdc125 Cysltr1 Adgrl3 Astn1 Osgin1 Itga4 Cd300a Gcnt1 Myo1g Dnaaf4 Apod Gfra1	Tgfb2 Thbs4 Ndrg4 Fgf18 Grin2c Tnfrsf12a Hbegf Arc Rnf165 Rcan1 Sph k1 Has2 Egr1 Megf10 Csf3r Msx2 Tnfaip6 Ppbp Rnd1 Thbs1 Ch25h Selp Cxcl1 Ccl7 Ccl2
Localization of cell	45	1,636	2	2.08E-03	Dnah6 Cd24a Rsph9 Adam8 Itgax Ifitm1 Itgb7 Rgn Wdr66 Gli1 Ddx4 Ptpn22 Ccdc125 Adgrl3 Astn1 Osgin1 Itga4 Cd300a Gcnt1 Myo1g Dnaaf4 Apod Gfra1	Tgfb2 Thbs4 Ndrg4 Fgf18 Tnfrsf12a Hbegf Arc Sphk1 Has2 Egr1 Megf1 0 Csf3r Msx2 Tnfaip6 Ppbp Rnd1 Thbs1 Ch25h Selp Cxcl1 Ccl7 Ccl2
Cell motility	45	1,636	2	2.08E-03	Dnah6 Cd24a Rsph9 Adam8 Itgax Ifitm1 Itgb7 Rgn Wdr66 Gli1 Ddx4 Ptpn22 Ccdc125 Adgrl3 Astn1 Osgin1 Itga4 Cd300a Gcnt1 Myo1g Dnaaf4 Apod Gfra1	Tgfb2 Thbs4 Ndrg4 Fgf18 Tnfrsf12a Hbegf Arc Sphk1 Has2 Egr1 Megf10 Csf3r Msx2 Tnfaip6 Ppbp Rnd1 Thbs1 Ch25h Selp Cxcl1 Ccl7 Ccl2
Immune response	45	1,619	2.1	2.08E-03	Cd24a Adam8 Tspan32 Itgax Il7r Ifitm1 Gbp10 Cd4 Ptpn22 Cysltr1 Naip6 Cd180 Nlrp1b Ciita Oasl1 Usp18 Naip5 Cd300a Rtp4 Oasl2 Il2ra Tap1 Rsad2 Fcer2a Apoa2 Cd19 H2-Ob H2-DMb2 Btla Prkcb Ifi44 Myo1g	Plscr1 Sbno2 Arid5a Batf Bcl3 Ppbp Thbs1 Adamts4 Cxcl1 Ccl7 Ccl2 Ptx3 Tgfb2
Defense response	44	1,546	2.1	2.08E-03	Cd24a Adam8 Tspan32 Itgax Il7r Ifitm1 Gbp10 Cd4 Ptpn22 Cysltr1 Naip6 Cd180 Nlrp1b Ciita Oasl1 Usp18 Naip5 Cd300a Rtp4 Oasl2 Il2ra Tap1 Rsad2 Cyp26b1 Gm5431 Gm4951 Fanca Apod	Plscr1 Sbno2 Arid5a Batf Bcl3 Ppbp Thbs1 Adamts4 Cxcl1 Ccl7 Ccl2 Ptx3 Sphk1 Tnfaip6 Selp Socs3
Response to cytokine	31	922	2.5	2.08E-03	Cd24a Il7r Ifitm1 Gbp10 Gm5431 Cd4 Il21r Ciita Oasl1 Itga4 Usp18 Gm4951 Oasl2 Il2ra	Plscr1 Sbno2 Ptprn Sphk1 Has2 Egr1 Csf3r Csf2rb2 Ppbp Thbs1 Selp Socs3 Cxcl1 Ccl7 Ccl2
Cellular response to cytokine stimulus	29	811	2.6	2.08E-03	Cd24a Il7r Ifitm1 Gbp10 Gm5431 Cd4 Il21r Ciita Oasl1 Itga4 Usp18 Gm4951 Oasl2 Il2ra	Plscr1 Sbno2 Ptprn Sphk1 Has2 Egr1 Csf3r Csf2rb2 Ppbp Thbs1 Selp Socs3 Cxcl1 Ccl7 Ccl2
Leukocyte migration	18	356	3.7	2.08E-03	Cd24a Adam8 Itgb7 Ptpn22 Itga4 Cd300a Gcnt1 Myo1g Apod	Tgfb2 Thbs4 Csf3r Ppbp Ch25h Selp Cxcl1 Ccl7 Ccl2 Kif28
Microtubule-based movement	18	387	3.4	2.40E-03	Dnah12 Dnah6 Cfap100 Rsph9 Kif19a Kif9 Rgn Wdr66 Kif15 Stk36 Ddx4 Dnah17 Dnah2 Dync2li1 Hap1 Dync2h1 Dnaaf4	Kif28
Response to external biotic stimulus	40	1,403	2.1	2.40E-03	Cyp1a1 Cd24a Adam8 Tspan32 Itgax Il7r Ifitm1 Naaladl2 Gbp10 Cd4 Ptpn22 Naip6 Cd52 Ifi44 Cd180 Nlrp1b Ciita Oasl1 Usp18 Naip5 Rtp4 Amy1 Oasl2 Tap1 Wdfy4 Rsad2 Slfn2	Plscr1 Sbno2 Arid5a Batf Bcl3 Ppbp Nos1 Fos Adamts4 Cxcl1 Ccl7 Ccl2 Ptx3
Response to other organism	40	1,400	2.1	2.40E-03	Cyp1a1 Cd24a Adam8 Tspan32 Itgax Il7r Ifitm1 Naaladl2 Gbp10 Cd4 Ptpn22 Naip6 Cd52 Ifi44 Cd180 Nlrp1b Ciita Oasl1 Usp18 Naip5 Rtp4 Amy1 Oasl2 Tap1 Wdfy4 Rsad2 Slfn2	Plscr1 Sbno2 Arid5a Batf Bcl3 Ppbp Nos1 Fos Adamts4 Cxcl1 Ccl7 Ccl2 Ptx3
Inflammatory response	25	698	2.7	3.52E-03	Cd24a Cyp26b1 Adam8 Cysltr1 Naip6 Cd180 Nlrp1b Ciita Usp18 Naip5 Cd300a Fanca Il2ra Apod	Plscr1 Sbno2 Sphk1 Tnfaip6 Ppbp Thbs1 Selp Socs3 Cxcl1 Ccl7 Ccl2
Response to biotic stimulus	40	1,439	2.1	3.68E-03	Cyp1a1 Cd24a Adam8 Tspan32 Itgax Il7r Ifitm1 Naaladl2 Gbp10 Cd4 Ptpn22 Naip6 Cd52 Ifi44 Cd180 Nlrp1b Ciita Oasl1 Usp18 Naip5 Rtp4 Amy1 Oasl2 Tap1 Wdfy4 Rsad2 Slfn2	Plscr1 Sbno2 Arid5a Batf Bcl3 Ppb p Nos1 Fos Adamts4 Cxcl1 Ccl7 Ccl2 Ptx3
Anatomical structure formation involved in morphogenesis	34	1,144	2.2	3.97E-03	Scgb3a1 Aldh1a1 Adam8 Itgax Ifitm1 E2f2 Cysltr1 Thbs2 Prkcb Itga4 Anpep	Tgfb2 Relt Myom2 Sall4 Synpo2l Sbno2 Thbs4 Kbtbd8 Fgf18 Tnfrsf12a Tnnt1 Junb Hbegf Dusp5 Sphk1 C sf3r Msx2 Aldh1a2 Esm1 Bcl3 Acta1 Thbs1 Ccl2
Cytokine-mediated signaling pathway	18	412	3.2	3.97E-03	Cd24a Il7r Ifitm1 Cd4 Il21r Oasl1 Usp18 Oasl2 Il2ra	Ptprn Sphk1 Egr1 Csf3r Csf2rb2 Ppbp Cxcl1 Ccl7 Ccl2
Biological process involved in interspecies interaction between organism	41	1,524	2	4.95E-03	Cyp1a1 Fcer2a Cd24a Adam8 Tspan32 Itgax Il7r Ifitm1 Naaladl2 Gbp10 Cd4 Ptpn22 Naip6 Cd52 Ifi44 Cd180 Nlrp1b Ciita Oasl1 Usp18 Naip5 Rtp4 Amy1 Oasl2 Tap1 Wdfy4 Rsad2 Slfn2	Plscr1 Sbno2 Arid5a Batf Bcl3 Ppbp Nos1 Fos Adamts4 Cxcl1 Ccl7 Ccl2 Ptx3
Response to bacterium	25	745	2.5	7.37E-03	Cyp1a1 Cd24a Il7r Naaladl2 Gbp10 Cd4 Ptpn22 Naip6 Cd52 Ifi44 Cd180 Usp18 Naip5 Amy1 Slfn2	Plscr1 Sbno2 Arid5a Bcl3 Ppbp Nos1 Fos Adamts4 Cxcl1 Ccl2
Cell mgration	39	1,474	2.0	9.29E-03	Cd24a Adam8 Itgax Ifitm1 Itgb7 Gli1 Ptpn22 Adgrl3 Astn1 Osgin1 Itga4 Cd300a Gcnt1 Myo1g Dnaaf4 Apod Gfra1	Tgfb2 Thbs4 Ndrg4 Fgf18 Tnfrsf12a Hbegf Arc Sphk1 Has2 Egr1 Megf10 Csf3r Msx2 Tnfaip6 Ppbp Rnd1 Thbs1 Ch2 5 h Selp Cxcl1 Ccl7 Ccl2
Cell adhesion	37	1,362	2	9.29E-03	Cd24a Cdhr4 Cdhr3 Adam8 Tspan32 Itgax Il7r Itgb7 Rasal3 Cd4 Ptpn22 Thbs2 Parvg Adgrl3 Astn1 Itga4 Cd300a Gcnt1 Myo1g Sspo Il2ra Apod	Tgfb2 Thbs4 Plaur Tnfrsf12a Has2 Megf10 Csf3r Tnfaip6 Lrrc4b Abat Rnd1 Thbs1 Sel e Selp Ccl2
Positive regulation of cell-cell adhesion	13	253	3.8	9.29E-03	Cd24a Adam8 Il7r Rasal3 Cd4 Ptpn22 Itga4 Il2ra	Plaur Has2 Megf10 Selp Ccl2

Genes (*n*) refers to number of differentially expressed genes in the specified pathway. Pathway genes (*n*) refers to the total number of genes in the specified pathway. FDR, false discovery rate; HFHS, high-fat, high-sucrose. *n* = 3 mice/group.

**Table 11.** ShinyGO KEGG pathway analysis showing up- and downregulated genes in male cardiac tissues in response to 4-mo HFHS feeding

Pathway	Genes (*n*)	Pathway Genes (*n*)	Fold Enrichment	Enrichment FDR	Upregulated Genes (vs. Normal Chow Diet)	Downregulated Genes (vs. Normal Chow Diet)
Hematopoietic cell lineage	11	92	8.8	1.11E-05	Il7r Fcer2a Cd4 Il2ra Itga4 Cd19 H2-DMb2 Anpep H2-Ob Cd24a	Csf3r
Cell adhesion molecules	11	160	5.1	9.43E-04	Itgb7 Cd4 Itga4 Siglec1 H2-DMb2 H2-Ob Selplg H2-Q6	Selp Sele Lrrc4b
Malaria	7	55	9.4	9.43E-04	Thbs2	Thbs4 Selp Sele Ccl2 Tgfb2 Thbs1
TNF signaling pathway	8	113	5.2	6.62E-03	Gm5431	Fos Sele Cxcl1 Ccl2 Junb Socs3 Bcl3
Cytokine-cytokine receptor interaction	13	289	3.3	6.62E-03	I17r Cd4 I12ra Il21r	Relt Tnfrsfl2a Csf3r Ppbp Cxcl1 Ccl7 Ccl2 Tgfb2 Csf2rb2
Primary immune-deficiency	5	36	10.3	6.62E-03	Il7r Ciita Cd4 Cd19 Tap1	
Leishmaniasis	6	69	6.4	1.15E-02	Itga4 H2-DMb2 H2-Ob Prkcb	Fos Tgfb2
Metabolism of xenobiotics by cytochrome P450	6	73	6.1	1.33E-02	Aldh3a1 Cbr3 Aldh3b1 Cbr2 Gsta3 Cyp1a1	
Phagosome	9	169	3.9	1.33E-02	Thbs2 Tap1 H2-DMb2 H2-Ob Dync2h1 H2-Q6	Thbs4 Nos1 Thbs1
Antigen processing and presentation	6	80	5.5	1.78E-02	Ciita Cd4 Tap1 H2-DMb2 H2-Ob H2-Q6	
Rheumatoid arthritis	6	86	5.2	2.37E-02	H2-DMb2 H2-Ob	Fos Cxcl1 Ccl2 Tgfb2
Human T-cell leukemia virus 1 infection	10	240	3.1	3.27E-02	E2f2 Cd4 Il2ra H2-DMb2 H2-Ob H2-Q6	Fos Msx2 Egr1 Tgfb2
Circadian entrainment	6	98	4.5	3.93E-02	Gucy1a2 Prkcb	Grin2c Fos Gnb3 Nos1
Intestinal immune network for IgA production	4	42	7	4.13E-02	Itgb7 Itga4 H2-DMb2 H2-Ob	
Th17 cell differentiation	6	104	4.3	4.59E-02	Cd4 I12ra Il21r H2-DMb2 H2-Ob	Fos

Genes (*n*) refers to number of differentially expressed genes in the specified pathway. Pathway genes (*n*) refers to the total number of genes in the specified pathway. FDR, false discovery rate; HFHS, high-fat, high-sucrose. *n* = 3 mice/group.

**Table 12.** GSEA enrichment analysis (Biological Processes) on male cardiac tissues in response to 4-mo HFHS feeding

Name	NES	FDR q-Val
GOBP_ANTIGEN_PROCESSING_AND_PRESENTATION_OF_PEPTIDE_ANTIGEN	2.0	0.035
GOBP_SPINDLE_CHECKPOINT_SIGNALING	2.0	0.035
GOBP_ANTIGEN_PROCESSING_AND_PRESENTATION_VIA_MHC_CLASS_IB	2.0	0.036
GOBP_REGULATION_OF_LYMPHOCYTE_MEDIATED_IMMUNITY	1.9	0.036
GOBP_LEUKOCYTE_MEDIATED_CYTOTOXICITY	1.9	0.037
GOBP_AXONEME_ASSEMBLY	1.9	0.038
GOBP_MITOTIC_SISTER_CHROMATID_SEPARATION	1.9	0.038
GOBP_REGULATION_OF_T_CELL_MEDIATED_CYTOTOXICITY	1.9	0.039
GOBP_ANTIGEN_PROCESSING_AND_PRESENTATION_OF_EXOGENOUS_PEPTIDE_ANTIGEN_VIA_MHC_CLASS_II	1.9	0.040
GOBP_TERPENOID_METABOLIC_PROCESS	2.0	0.041
GOBP_SENSORY_PERCEPTION_OF_CHEMICAL_STIMULUS	1.9	0.041
GOBP_ANTIGEN_PROCESSING_AND_PRESENTATION_OF_ENDOGENOUS_ANTIGEN	1.9	0.042
GOBP_REGULATION_OF_MAMMARY_GLAND_EPITHELIAL_CELL_PROLIFERATION	1.9	0.043
GOBP_REGULATION_OF_MITOTIC_SISTER_CHROMATID_SEGREGATION	2.0	0.046
GOBP_LIPID_CATABOLIC_PROCESS	1.9	0.046
GOBP_CELLULAR_LIPID_CATABOLIC_PROCESS	1.9	0.049
GOBP_EXTRACELLULAR_TRANSPORT	1.9	0.049
GOBP_MITOCHONDRIAL_TRANSLATION	−2.5	0
GOBP_RIBOSOME_BIOGENESIS	−2.4	0
GOBP_MITOCHONDRIAL_GENE_EXPRESSION	−2.4	0
GOBP_RIBONUCLEOPROTEIN_COMPLEX_BIOGENESIS	−2.4	0
GOBP_RRNA_METABOLIC_PROCESS	−2.3	0
GOBP_NCRNA_PROCESSING	−2.3	0
GOBP_MITOCHONDRIAL_RESPIRATORY_CHAIN_COMPLEX_ASSEMBLY	−2.2	0
GOBP_PROTON_MOTIVE_FORCE_DRIVEN_ATP_SYNTHESIS	−2.2	0
GOBP_TRANSLATION_AT_SYNAPSE	−2.2	0
GOBP_NADH_DEHYDROGENASE_COMPLEX_ASSEMBLY	−2.2	0
GOBP_RIBOSOMAL_SMALL_SUBUNIT_BIOGENESIS	−2.2	0
GOBP_RIBOSOMAL_LARGE_SUBUNIT_BIOGENESIS	−2.2	0
GOBP_MATURATION_OF_LSU_RRNA	−2.2	0
GOBP_CYTOPLASMIC_TRANSLATION	−2.1	0
GOBP_MATURATION_OF_SSU_RRNA	−2.1	0.004
GOBP_REGULATION_OF_MITOCHONDRIAL_GENE_EXPRESSION	−2.1	0.005
GOBP_TRNA_PROCESSING	−2.0	0.007
GOBP_RNA_MODIFICATION	−2.0	0.007
GOBP_MATURATION_OF_LSU_RRNA_FROM_TRICISTRONIC_RRNA_TRANSCRIPT_SSU_RRNA_5_8S_RRNA_LSU_RRNA	−2.0	0.009
GOBP_REGULATION_OF_MITOCHONDRIAL_TRANSLATION	−2.0	0.014
GOBP_TRNA_MODIFICATION	−2.0	0.013
GOBP_POSITIVE_REGULATION_OF_MITOCHONDRIAL_TRANSLATION	−2.0	0.015
GOBP_SARCOMERE_ORGANIZATION	−1.9	0.017
GOBP_MATURATION_OF_SSU_RRNA_FROM_TRICISTRONIC_RRNA_TRANSCRIPT_SSU_RRNA_5_8S_RRNA_LSU_RRNA	−1.9	0.017
GOBP_PSEUDOURIDINE_SYNTHESIS	−1.9	0.018

FDR, false discovery rate; GSEA, Gene set enrichment analysis; HFHS, high-fat, high-sucrose; NES, normalized enrichment score. *n* = 3 mice/group.

**Table 13.** GSEA enrichment analysis (Canonical Pathways) on male cardiac tissues in response to 4-mo HFHS feeding

Name	NES	FDR q-Val
REACTOME_IMMUNOREGULATORY_INTERACTIONS_BETWEEN_A_LYMPHOID_AND_A_NON_LYMPHOID_CELL	2.1	0.005
WP_GLUTATHIONE_METABOLISM	2.0	0.009
REACTOME_BIOLOGICAL_OXIDATIONS	2.0	0.011
WP_RETINOL_METABOLISM	2.0	0.016
REACTOME_DRUG_ADME	1.9	0.015
REACTOME_PLASMA_LIPOPROTEIN_ASSEMBLY_REMODELING_AND_CLEARANCE	1.9	0.016
WP_METAPATHWAY_BIOTRANSFORMATION	1.9	0.017
REACTOME_PHASE_I_FUNCTIONALIZATION_OF_COMPOUNDS	1.9	0.019
REACTOME_ER_PHAGOSOME_PATHWAY	1.9	0.019
REACTOME_CDC42_GTPASE_CYCLE	1.9	0.019
REACTOME_HOMOLOGOUS_DNA_PAIRING_AND_STRAND_EXCHANGE	1.8	0.033
REACTOME_RESOLUTION_OF_D_LOOP_STRUCTURES	1.8	0.050
REACTOME_RAC1_GTPASE_CYCLE	1.8	0.048
WP_MICROGLIA_PATHOGEN_PHAGOCYTOSIS_PATHWAY	1.8	0.047
REACTOME_TRANSLATION	−2.6	0
REACTOME_MITOCHONDRIAL_TRANSLATION	−2.5	0
REACTOME_MAJOR_PATHWAY_OF_RRNA_PROCESSING_IN_THE_NUCLEOLUS_AND_CYTOSOL	−2.5	0
REACTOME_NONSENSE_MEDIATED_DECAY_NMD_INDEPENDENT_OF_THE_EXON_JUNCTION_COMPLEX_EJC	−2.3	0
REACTOME_EUKARYOTIC_TRANSLATION_INITIATION	−2.3	0
REACTOME_FORMATION_OF_A_POOL_OF_FREE_40S_SUBUNITS	−2.3	0
WP_CYTOPLASMIC_RIBOSOMAL_PROTEINS	−2.3	0
REACTOME_COMPLEX_I_BIOGENESIS	−2.2	0
WP_ELECTRON_TRANSPORT_CHAIN	−2.2	0
REACTOME_RESPIRATORY_ELECTRON_TRANSPORT	−2.1	0
REACTOME_RESPIRATORY_ELECTRON_TRANSPORT_ATP_SYNTHESIS_BY_CHEMIOSMOTIC_COUPLING_AND_HEAT_PRODUCTION_BY_UNCOUPLING_PROTEINS	−2.1	0
REACTOME_NONSENSE_MEDIATED_DECAY_NMD	−2.0	0.005
REACTOME_ACTIVATION_OF_THE_MRNA_UPON_BINDING_OF_THE_CAP_BINDING_COMPLEX_AND_EIFS_AND_SUBSEQUENT_BINDING_TO_43S	−2.0	0.005
WP_OXIDATIVE_PHOSPHORYLATION	−1.9	0.010
REACTOME_CARBOXYTERMINAL_POST_TRANSLATIONAL_MODIFICATIONS_OF_TUBULIN	−1.9	0.012
REACTOME_PROCESSING_OF_CAPPED_INTRON_CONTAINING_PRE_MRNA	−1.9	0.013
REACTOME_MRNA_SPLICING	−1.9	0.027
REACTOME_STRIATED_MUSCLE_CONTRACTION	−1.8	0.031
REACTOME_THE_CITRIC_ACID_TCA_CYCLE_AND_RESPIRATORY_ELECTRON_TRANSPORT	−1.8	0.047
REACTOME_SEALING_OF_THE_NUCLEAR_ENVELOPE_NE_BY_ESCRT_III	−1.8	0.048
REACTOME_MITOCHONDRIAL_BIOGENESIS	−1.8	0.047

FDR, false discovery rate; GSEA, Gene set enrichment analysis; HFHS, high-fat, high-sucrose; NES, normalized enrichment score. *n* = 3 mice/group.

**Table 14.** ShinyGO Gene Ontology Biological Processes analysis showing up- and down-regulated genes in female cardiac tissues in response to 4 months HFHS feeding

Pathway	Genes (*n*)	Pathway Genes (*n*)	Fold Enrichment	Enrichment FDR	Upregulated Genes (vs. Normal Chow Diet)	Downregulated Genes (vs. Normal Chow Diet)
Regulation of ribonuclease activity	5	15	33.8	8.27E-04	Oasl2 Oas3 Oas2 Oasl1 Oas1g	
Defense response to symbiont	13	254	5.2	1.74E-03	Oas3 Zbp1 Oas1g Oasl1 Oas2 Ifit3 Oasl2 Irf7 Ifit3b Slfn8 Ifit1 Isg20	Tlr9
Defense response to virus	13	254	5.2	1.74E-03	Oas3 Zbp1 Oas1g Oasl1 Oas2 Ifit3 Oasl2 Irf7 Ifit3b Slfn8 Ifit1 Isg20	Tlr9
Positive regulation of calcium ion import	5	26	19.5	3.71E-03	Atp2a1 Lgals3 Adrb2	Agtr1a Cxcl12
Response to virus	14	330	4.3	3.71E-03	Cyp1a1 Oas3 Zbp1 Oas1g Oasl1 Oas2 Ifit3 Oasl2 Irf7 Ifit3b Slfn8 Ifit1 Isg20	Tlr9
Regulation of nuclease activity	5	28	18.1	4.06E-03	Oasl2 Oas3 Oas2 Oasl1 Oaslg	
Defense response	34	1,546	2.2	4.23E-03	Lgals3 Ccl8 Oas3 Zbp1 Ifi206 Oas1g Cd180 Wfdc3 Cysltr1 Oasl1 Gm4951 Grm7 Cd300a Fanca Oas2 Cers6 Ifit3 Ifi213 Oasl2 Irf7 Il1rap Lbp Naip5 Ifit3b Adrb2 Serpine1 Slfn8 Ifit1 Isg20	Lpl Agtr1a Cxcl12 Tlr9 Stab2
Negative regulation of viral genome replication	6	53	11.5	5.61E-03	Oasl2 Oas3 Oas2 Oasl1 Oaslg Isg20	
Toxin metabolic process	4	17	23.8	6.63E-03	Pam Cyp1a1 Fmo2	Ddc
Interleukin-27-mediated signaling pathway	3	6	50.7	6.63E-03	Oasl2 Oas2 Oasl1	
l-lysine transport	3	7	43.4	8.97E-03	Agt	Slc7a1 Slc25a29
l-lysine transmembrane transport	3	7	43.4	8.97E-03	Agt	Slc7a1 Slc25a29
Innate immune response	20	742	2.7	1.28E-02	Lgals3 Ccl8 Oas3 Zbp1 Ifi206 Oas1g Cd180 Wfdc3 Oasl1 Oas2 Ifit3 Ifi213 Oasl2 Irf7 Il1rap Lbp Naip5 Ifit1 Isg20	Tlr9
Defense response to other organism	24	1,012	2.4	1.59E-02	Lgals3 Ccl8 Oas3 Zbp1 Ifi206 Oas1g Cd180 Wfdc3 Oasl1 Oas2 Ifit3 Ifi213 Oasl2 Irf7 Il1rap Lbp Naip5 Ifit3b Serpine1 Slfn8 Ifit1 Isg20	Tlr9 Stab2
Retina layer formation	4	24	16.9	1.66E-02	Megfl1 Fat3 Sdk2 Sdk1	
l-arginine transmembrane transport	3	9	33.8	1.66E-02	Agt	Slc7a1 Slc25a29
Response to biotic stimulus	30	1,439	2.1	1.66E-02	Cyp1a1 Lgals3 Ccl8 Oas3 Zbp1 Ifi206 Oas1g Cd180 Wfdc3 Ggt7 Oasl1 Oas2 Ifit3 Ifi213 Oasl2 Irf7 Il1rap Lbp Txnip Naip5 Ifit3b Ifi44 Serpine1 Slfn8 Ifit1 Isg20	Lpl Cxcl12 Tlr9 Stab2
Arginine transmembrane transport	3	10	30.4	1.90E-02	Agt	Slc7a1 Slc25a29
Cellular response to interferon-beta	5	48	10.6	1.90E-02	Ifit1 Ifi206 Ifi213 Gm4951 Ifit3	
Response to external biotic stimulus	29	1,403	2.1	1.97E-02	Cyp1a1 Lgals3 Ccl8 Oas3 Zbp1 Ifi206 Oas1g Cd180 Wfdc3 Ggt7 Oasl1 Oas2 Ifit3 Ifi213 Oasl2 Irf7 Il1rap Lbp Naip5 Ifit3b Ifi44 Serpine1 Slfn8 Ifit1 Isg20	Lpl Cxcl12 Tlr9 Stab2

Genes (*n*) refers to number of differentially expressed genes in the specified pathway. Pathway genes (*n*) refers to the total number of genes in the specified pathway. FDR, false discovery rate; HFHS, high-fat, high-sucrose. *n* = 3 mice/group.

**Table 15.** GSEA enrichment analysis (Biological Processes) on female cardiac tissues in response to 4-mo HFHS feeding

Name	NES	FDR q-Val
GOBP_TERPENOID_METABOLIC_PROCESS	2.0	0.009
GOBP_COLLAGEN_FIBRIL_ORGANIZATION	2.0	0.007
GOBP_EXTERNAL_ENCAPSULATING_STRUCTURE_ORGANIZATION	2.0	0.004
GOBP_ASTROCYTE_DEVELOPMENT	2.0	0.003
GOBP_OLEFINIC_COMPOUND_METABOLIC_PROCESS	2.0	0.005
GOBP_EXTRACELLULAR_MATRIX_ASSEMBLY	2.0	0.009
GOBP_NEGATIVE_REGULATION_OF_VIRAL_PROCESS	1.9	0.012
GOBP_LONG_CHAIN_FATTY_ACID_METABOLIC_PROCESS	1.9	0.022
GOBP_NEGATIVE_REGULATION_OF_PEPTIDASE_ACTIVITY	1.9	0.024
GOBP_ENDOLYSOSOMAL_TOLL_LIKE_RECEPTOR_SIGNALING_PATHWAY	1.9	0.023
GOBP_BONE_MINERALIZATION	1.9	0.022
GOBP_ANTIGEN_PROCESSING_AND_PRESENTATION_OF_ENDOGENOUS_ANTIGEN	1.9	0.025
GOBP_ASTROCYTE_ACTIVATION	1.9	0.024
GOBP_NEGATIVE_REGULATION_OF_VIRAL_GENOME_REPLICATION	1.9	0.027
GOBP_PRIMARY_ALCOHOL_METABOLIC_PROCESS	1.9	0.026
GOBP_NEGATIVE_REGULATION_OF_RESPONSE_TO_CYTOKINE_STIMULU	1.8	0.029
GOBP_CELL_SUBSTRATE_ADHESION	1.8	0.037
GOBP_REGULATION_OF_BIOMINERAL_TISSUE_DEVELOPMENT	1.8	0.035
GOBP_SYNAPTIC_TRANSMISSION_GLUTAMATERGIC	1.8	0.035
GOBP_ANTIGEN_PROCESSING_AND_PRESENTATION_VIA_MHC_CLASS_IB	1.8	0.034
GOBP_POSITIVE_REGULATION_OF_T_CELL_MEDIATED_IMMUNITY	1.8	0.034
GOBP_COMPLEMENT_ACTIVATION	1.8	0.034
GOBP_METANEPHROS_MORPHOGENESIS	1.8	0.032
GOBP_REGULATION_OF_BONE_MINERALIZATION	1.8	0.031
GOBP_CELL_DIFFERENTIATION_INVOLVED_IN_METANEPHROS_DEVELOP	1.8	0.031
GOBP_PROTON_MOTIVE_FORCE_DRIVEN_ATP_SYNTHESIS	−3.1	0
GOBP_MITOCHONDRIAL_TRANSLATION	−3.1	0
GOBP_OXIDATIVE_PHOSPHORYLATION	−3.1	0
GOBP_AEROBIC_RESPIRATION	−3.0	0
GOBP_MITOCHONDRIAL_RESPIRATORY_CHAIN_COMPLEX_ASSEMBLY	−3.0	0
GOBP_MITOCHONDRIAL_GENE_EXPRESSION	−2.9	0
GOBP_NADH_DEHYDROGENASE_COMPLEX_ASSEMBLY	−2.9	0
GOBP_ATP_SYNTHESIS_COUPLED_ELECTRON_TRANSPORT	−2.9	0
GOBP_AEROBIC_ELECTRON_TRANSPORT_CHAIN	−2.9	0
GOBP_MITOCHONDRIAL_ELECTRON_TRANSPORT_NADH_TO_UBIQUINO	−2.5	0
GOBP_CELLULAR_RESPIRATION	−2.4	0
GOBP_ATP_BIOSYNTHETIC_PROCESS	−2.4	0
GOBP_NUCLEOSIDE_TRIPHOSPHATE_BIOSYNTHETIC_PROCESS	−2.4	0
GOBP_REGULATION_OF_MITOCHONDRIAL_TRANSLATION	−2.3	0
GOBP_ELECTRON_TRANSPORT_CHAIN	−2.3	0
GOBP_TRICARBOXYLIC_ACID_CYCLE	−2.3	0
GOBP_INNER_MITOCHONDRIAL_MEMBRANE_ORGANIZATION	−2.2	0
GOBP_REGULATION_OF_MITOCHONDRIAL_GENE_EXPRESSION	−2.2	0.001
GOBP_BRANCHED_CHAIN_AMINO_ACID_CATABOLIC_PROCESS	−2.2	0.001
GOBP_PROTEIN_TARGETING_TO_MITOCHONDRION	−2.2	0.001
GOBP_PROTEIN_LOCALIZATION_TO_MITOCHONDRION	−2.2	0.001
GOBP_MITOCHONDRIAL_RNA_METABOLIC_PROCESS	−2.2	0.001
GOBP_RELEASE_OF_SEQUESTERED_CALCIUM_ION_INTO_CYTOSOL_BY_ENDOPLASMIC_RETICULUM	−2.1	0.003
GOBP_ESTABLISHMENT_OF_PROTEIN_LOCALIZATION_TO_MITOCHONDRIAL_MEMBRANE	−2.1	0.003
GOBP_CRISTAE_FORMATION	−2.1	0.003

FDR, false discovery rate; GSEA, Gene set enrichment analysis; HFHS, high-fat, high-sucrose; NES, normalized enrichment score. *n* = 3 mice/group.

**Table 16.** GSEA enrichment analysis (Canonical Pathways) on female cardiac tissues in response to 4-mo HFHS feeding

Name	NES	FDR q-Val
REACTOME_EXTRACELLULAR_MATRIX_ORGANIZATION	2.1	0.000
REACTOME_IMMUNOREGULATORY_INTERACTIONS_BETWEEN_A_LYMPHOID_AND_A_NON_LYMPHOID_CELL	2.0	0.000
REACTOME_PHASE_I_FUNCTIONALIZATION_OF_COMPOUNDS	2.0	0.001
REACTOME_DEGRADATION_OF_THE_EXTRACELLULAR_MATRIX	2.0	0.002
REACTOME_CYTOCHROME_P450_ARRANGED_BY_SUBSTRATE_TYPE	1.9	0.008
REACTOME_COLLAGEN_FORMATION	1.9	0.008
WP_PROSTAGLANDIN_SYNTHESIS_AND_REGULATION	1.9	0.007
REACTOME_COLLAGEN_BIOSYNTHESIS_AND_MODIFYING_ENZYMES	1.9	0.007
REACTOME_INTEGRIN_CELL_SURFACE_INTERACTIONS	1.9	0.006
REACTOME_SPHINGOLIPID_METABOLISM	1.9	0.007
BIOCARTA_EICOSANOID_PATHWAY	1.9	0.007
REACTOME_COLLAGEN_CHAIN_TRIMERIZATION	1.8	0.009
REACTOME_ARACHIDONIC_ACID_METABOLISM	1.8	0.026
REACTOME_ELASTIC_FIBER_FORMATION	1.8	0.027
REACTOME_MOLECULES_ASSOCIATED_WITH_ELASTIC_FIBRES	1.8	0.029
REACTOME_ECM_PROTEOGLYCANS	1.8	0.034
REACTOME_ASSEMBLY_OF_COLLAGEN_FIBRILS_AND_OTHER_MULTIMERIC_STRUCTURES	1.8	0.035
WP_TYROBP_CAUSAL_NETWORK_IN_MICROGLIA	1.7	0.042
REACTOME_COLLAGEN_DEGRADATION	1.7	0.050
REACTOME_BIOLOGICAL_OXIDATIONS	1.7	0.049
WP_OXIDATION_BY_CYTOCHROME_P450	1.7	0.047
REACTOME_SPHINGOLIPID_DE_NOVO_BIOSYNTHESIS	1.7	0.049
REACTOME_RESPIRATORY_ELECTRON_TRANSPORT	−3.1	0.000
REACTOME_MITOCHONDRIAL_TRANSLATION	−3.0	0.000
REACTOME_COMPLEX_I_BIOGENESIS	−2.9	0.000
REACTOME_THE_CITRIC_ACID_TCA_CYCLE_AND_RESPIRATORY_ELECTRON_TRANSPORT	−2.8	0.000
WP_OXIDATIVE_PHOSPHORYLATION	−2.8	0.000
REACTOME_RESPIRATORY_ELECTRON_TRANSPORT_ATP_SYNTHESIS_BY_CHEMIOSMOTIC_COUPLING_AND_HEAT_PRODUCTION_BY_UNCOUPLING_PROTEINS	−2.8	0.000
REACTOME_TRANSLATION	−2.7	0.000
WP_ELECTRON_TRANSPORT_CHAIN	−2.6	0.000
REACTOME_BRANCHED_CHAIN_AMINO_ACID_CATABOLISM	−2.5	0.000
REACTOME_CITRIC_ACID_CYCLE_TCA_CYCLE	−2.4	0.000
REACTOME_FORMATION_OF_ATP_BY_CHEMIOSMOTIC_COUPLING	−2.3	0.000
REACTOME_MITOCHONDRIAL_BIOGENESIS	−2.3	0.000
REACTOME_GLYOXYLATE_METABOLISM_AND_GLYCINE_DEGRADATION	−2.1	0.005
REACTOME_PYRUVATE_METABOLISM_AND_CITRIC_ACID_TCA_CYCLE	−2.0	0.009
WP_GLYCOLYSIS_AND_GLUCONEOGENESIS	−1.9	0.017

FDR, false discovery rate; GSEA, Gene set enrichment analysis; HFHS, high-fat, high-sucrose; NES, normalized enrichment score. *n* = 3 mice/group.

**Figure 9.**
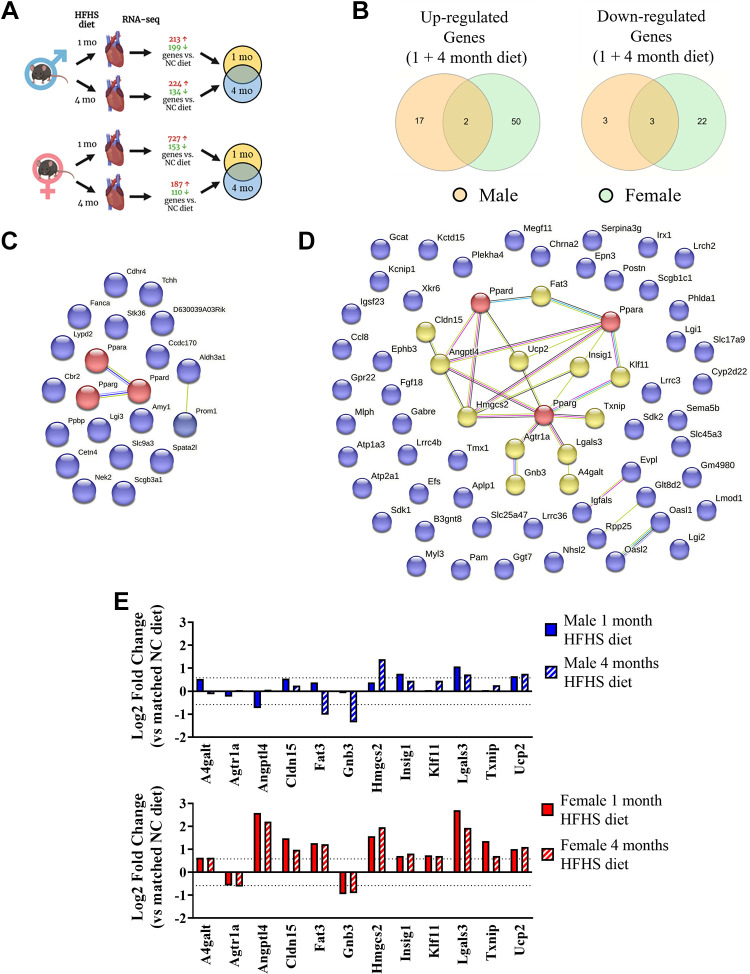
RNA-sequencing analysis of genes similarly regulated in male and female hearts after 1- and 4-mo exposure to high-fat/high-sucrose (HFHS) diet. *A*: overview of RNA-seq analysis workflow. Generated using Biorender.com. *B*: Venn diagram showing the overlap between up- and downregulated genes similarly regulated across 1- and 4-mo exposures to HFHS diet in male versus female mice. *C*: STRING protein-protein interaction network using Ppar genes (Red) as “bait,” to determine predicted interactions with genes uniquely regulated in male hearts after 1- and 4-mo diet exposure (blue). *D*: STRING protein-protein interaction network using Ppar genes (Red) as “bait,” to determine predicted interactions with genes uniquely regulated in female hearts after 1- and 4-mo diet exposure (blue). Genes with significant predicted interactions with Ppar genes are shown in yellow. *E*: Log2 fold change versus age- and sex-matched normal chow diet group of genes with significant predicted interactions with Ppar genes identified in Fig. 5*D* are displayed for male hearts (*top*) and female hearts (*bottom*). *n* = 3 mice/group.

Next, we hypothesized that genes that were similarly regulated across the 1- and 4-mo diet time points may be highly important in influencing the cardiac functional differences we observed after 4 mo of HFHS feeding in male and female mice. Gene expression was stratified according to sex and diet duration, and the overlap between the 1- and 4-mo gene lists was determined for each sex as illustrated in Fig. 9*A*. The overlap between the resulting lists was evaluated to discover genes commonly or uniquely regulated in male and female hearts across both time points; we found that most were unique to sex, with males showing 17/19 genes uniquely upregulated and 3/6 genes uniquely downregulated, and females showing 50/52 genes uniquely upregulated and 22/25 genes uniquely downregulated genes (Fig. 9*B*, Table 17). The gene lists identified many of the genes regulated at 1 and 4 mo of diet in female mice were related to PPAR signaling, as highlighted in the KEGG pathway analysis after 1 mo of HFHS diet. We performed STRING network analysis of protein-protein interactions relating to the PPAR pathway using *Ppara*, *Ppard*, and *Pparg* genes to interrogate our gene list. In male mice, we found no interactions between *Ppar* genes and our gene list (Fig. 9*C*), whereas interactions were identified between the *Ppar* genes and 12 genes from the female list (Fig. 9*D*). Genes from this network were plotted to visualize the difference in expression between male (Fig. 9*E*, *top*) and female (Fig. 9*E*, *bottom*) hearts after short- and long-term HFHS feeding. Data supplements can be accessed here: https://doi.org/10.5281/zenodo.8191388.

**Table 17.** Significantly up- or downregulated genes in male or female cardiac tissues across both 1- and 4-mo time points of HFHS feeding

	Male					Female			
	1-Mo Diet Consumption		4-Mo Diet Consumption			1-Mo Diet Consumption		4-Mo Diet Consumption	
Gene Symbol	Log2FC	*P* Value	Log2FC	*P* Value	Gene Symbol	Log2FC	*P* Value	Log2FC	*P* Value
*Upregulated Genes*					*Upregulated Genes*				
Prom1	1.8	0.004	1.8	0.003	Insig1	0.7	2.9E-06	0.8	6.6E-08
Scgb3a1	7.3	0.005	7.3	0.005	Txnip	1.4	3.3E-05	0.7	0.027
Cdhr4	2.0	0.008	2.4	0.001	Cldn15	1.5	7.3E-05	1.0	0.008
Spata2l	0.7	0.008	0.6	0.020	Nhsl2	0.7	2.1E-04	0.6	0.001
Amy1	0.6	0.010	0.7	0.003	Klf11	0.7	2.5E-04	0.7	3.9E-04
Cetn4	1.7	0.015	1.9	0.004	Slc17a9	1.4	0.001	1.0	0.018
Fanca	0.7	0.016	0.7	0.006	Angptl4	2.6	0.001	2.2	0.003
Cbr2	2.3	0.020	3.0	0.003	Lmod1	1.2	0.001	0.7	0.041
LOC102636360	1.2	0.020	1.4	0.004	Postn	1.0	0.002	0.7	0.016
Aldh3a1	2.7	0.022	2.6	0.024	Gabre	1.3	0.002	1.2	0.003
Lypd2	2.4	0.024	3.0	0.006	Gm11665	1.7	0.003	0.8	0.046
Tchh	1.8	0.026	1.6	0.029	Atp2a1	2.2	0.005	2.0	0.010
Nek2	1.3	0.026	1.5	0.010	Igsf23	2.2	0.006	1.5	0.045
Igkc	3.2	0.026	4.8	0.002	Lrch2	0.8	0.006	0.6	0.033
Ccdc170	1.6	0.028	1.5	0.029	Ccl8	1.7	0.006	1.8	0.004
Stk36	1.2	0.039	1.3	0.025	Glt8d2	1.0	0.007	1.1	0.023
D630039A03Rik	1.1	0.049	1.3	0.032	Lgals3	2.7	0.007	1.9	0.048
*Downregulated genes*					A4galt	0.6	0.008	0.6	0.007
Slc9a3	−1.6	0.012	−1.5	0.036	Sdk2	1.2	0.008	1.2	0.008
Lgi3	−0.9	0.017	−0.9	0.018	Oasl2	0.7	0.008	0.7	0.004
Ppbp	−1.4	0.046	−1.2	0.036	Sdk1	0.8	0.008	0.6	0.029
					Abhd1	0.9	0.009	0.7	0.033
					Gm34114	0.7	0.009	0.8	0.002
					Hmgcs2	1.6	0.011	2.0	0.002
					Xkr6	1.0	0.011	0.9	0.006
					Lrrc3	1.1	0.012	1.1	0.022
					Chrna2	1.4	0.014	1.2	0.045
					LOC118567636	1.5	0.015	1.9	0.004
					B3gnt8	0.6	0.015	0.6	0.024
					Atp1a3	1.0	0.015	1.2	0.007
					Mlph	1.7	0.020	1.8	0.017
					Tpbgl	0.8	0.022	0.7	0.031
					Aplp1	0.9	0.023	0.9	0.028
					Plekha4	0.7	0.024	0.9	0.003
					Megf11	1.4	0.027	1.5	0.018
					Ucp2	1.0	0.027	1.1	0.016
					Pam	1.4	0.027	1.3	0.047
					Ggt7	0.8	0.028	1.0	0.004
					Slc45a3	0.9	0.030	1.1	0.006
					Lrrc36	1.1	0.030	1.2	0.013
					Efs	0.7	0.031	0.7	0.020
					Cyp2d22	0.6	0.034	0.6	0.027
					Kctd15	0.8	0.036	0.8	0.019
					Oasl1	0.8	0.036	0.9	0.015
					Rpp25	1.1	0.040	1.2	0.029
					Serpina3g	1.3	0.040	2.6	1.5E-04
					Fat3	1.3	0.042	1.2	0.043
					Lgi2	0.9	0.044	1.2	0.007
					Evpl	1.6	0.045	1.9	0.028
					*Downregulated genes*				
					Tmx1	−0.7	7.4E-06	−0.8	1.2E-07
					Gcat	−0.6	1.6E-04	−0.7	5.3E-06
					Scgb1c1	−1.4	4.2E-04	−1.3	0.001
					Myl3	−0.8	4.2E-04	−0.6	0.006
					Fgf18	−0.8	0.002	−0.7	0.005
					Gm36670	−1.4	0.003	−1.5	0.002
					Phlda1	−0.8	0.005	−0.9	0.001
					Agtr1a	−0.6	0.006	−0.6	0.003
					Kcnip1	−2.1	0.011	−1.2	0.030
					Lrrc4b	−0.7	0.013	−1.0	2.2E-04
					Igfals	−0.9	0.020	−1.1	0.007
					Slc25a47	−0.6	0.024	−0.6	0.023
					Ephb3	−0.6	0.024	−0.7	0.014
					Irx1	−1.1	0.026	−1.0	0.044
					Gpr22	−1.1	0.027	−1.1	0.026
					Gm38585	−0.7	0.031	−0.9	0.013
					Gnb3	−1.0	0.032	−0.9	0.036
					Epn3	−0.6	0.033	−0.9	0.004
					Gm15246	−0.6	0.035	−0.7	0.022
					Gm30262	−1.0	0.038	−1.5	0.003
					Lgi1	−0.9	0.040	−0.9	0.040
					Sema5b	−0.8	0.045	−1.0	0.014

Log2FC, Log2 fold change compared with normal chow diet of the same sex and time point. *n* = 3 mice/group.

## DISCUSSION

In this study, our data show sex-based differences in the development of obesity and associated cardiometabolic changes across short- and longer-term “Western-style” obesogenic diet. Our systems-based approach to identify cardiac molecular differences between male and female mice fed 1 versus 4 mo of obesogenic HFHS diet showed *1*) predilection toward the development of cardiac dysfunction in males despite comparable metabolic dysfunction in both male and female mice; *2*) sex-dependent cardiac changes in gene expressions of m6A methylation machinery that correlates with changes in LVEF; and *3*) sex-based differential cardiac transcriptional changes. To date, molecular mechanisms underlying sex-based differences in the development of cardiac and metabolic changes over various obesogenic exposures are understudied. To our knowledge, this study represents the first in-depth longitudinal analysis of transcriptional differences and the potential role of epitranscriptomic changes in differentiating sex-based cardiac functional changes upon exposure to obesogenic diet of various duration.

Although comparable animal studies that investigate sex-dependent cardiac functional changes during diet-induced obesity are limited, favorable metabolic profiles in the female sex have been demonstrated in other animal studies (63–67). Our data show that while females also exhibited impaired metabolic functions after 1 and 4 mo of HFHS feeding, male mice were proportionately worse. We also demonstrated that metabolic perturbations in males were associated with detrimental cardiac changes and subsequent development of cardiac dysfunction, which was not observed in females. Similar to our previous studies (54, 55, 68), our current data show significant impairment in both systolic and diastolic function in male mice after longer-term feeding, consistent with metabolic heart disease pathophysiology (8), whereas female mice had a lack of cardiac dysfunction even with longer-term duration of feeding. These results are consistent with Louwe et al. (69) who showed a cardiac decline in males but not in female mice after 12 wk of a high fat-only obesogenic diet. These results suggest that independent of the type of obesogenic diet, females are protected against diet-induced cardiometabolic perturbations.

Our cardiac transcriptome and pathway analyses reveal stark sex differences in cardiac responses to obesogenic stress. Enriched pathways suggest males predominantly activate early metabolic remodeling, while an early protective cardiac remodeling response ensues in females. Joseph et al. (70) also demonstrated similar enriched pathways in male and female mouse hearts after short-term high-fat-only diet, suggesting some responses may be consistent across different diet formulations. Male responses are consistent with metabolic substrate utilization changes in the heart after as little as 2 wk of Western-style diet exposure (71, 72). Although pathological cardiac remodeling is well documented in longer-term obesity (73, 74), it is unknown whether adaptive cardiac remodeling is an early protective mechanism during obesity development in either sex, although reports have shown that sex-specific adaptive cardiac remodeling is protective in female mice against other cardiac insults (75, 76). Longer-term diet exposure resulted in a predominantly immunoregulatory response in both sexes, however, the limited overlap in the specific pathways suggests that the cardiac immune pathways may also respond in a sex-dependent manner during obesogenic stress.

The longitudinal analysis of diet-induced changes in cardiac gene expression in our study indicates that sex-specific subsets of genes are dysregulated over an extended period during obesity development. We observed sex differences in PPAR pathway genes both in the absence and presence of an obesogenic diet over time exclusively in female but not in male hearts, suggesting a role for this pathway in cardiac sex-dependent functional changes. The PPAR network has a critical role in regulating metabolism, inflammation, oxidative stress, and reportedly, biological functions in a sex-specific fashion (77–81). Nonetheless, our transcriptional data should be interpreted with caution, given that protein-level data remains largely unknown. We also note that the PPAR pathway was not identified in the enrichment analysis of both 1- and 4-mo time points in female hearts, however, the small number of genes regulated at 4 mo did not allow us to identify any significant KEGG pathways.

Epigenetic modifications, including histone, DNA methylation, and noncoding RNA molecules, have all been shown to regulate gene expression in various cardiometabolic disease processes (29, 82, 83). Accumulating evidence shows that m6A RNA methylation, the most common and abundant epigenetic modification in eukaryotic RNA, regulates obesity-related metabolic and cardiac pathophysiology (43, 84, 85). Fat mass and obesity-associated protein (FTO) is a pivotal regulator of adipogenesis and is a determinant of obesity development (31, 46, 47, 86–89). Although FTO and other components of the m6A RNA methylation machinery such as methyltransferase-like (METTL)3 and METTL14 have been implicated in cardiac pathophysiology (84, 85, 90, 91), sex-based differences in cardiac m6A machinery have not previously been described. Our data show that m6A machinery genes are dynamically regulated in the heart across short- and long-term exposure to obesogenic diet in males and correlate with left ventricular ejection fraction. Consistent with other literature in models of longer-term diet-induced obesity in male mice (85, 92, 93), we also observed a downregulation of *Fto* and *Mettl3* RNA expression, which correspond to a decline in cardiac systolic and diastolic function. Interestingly, there were no changes in any of the cardiac m6A machinery genes in female mice, irrespective of diet duration. Consistent with our observations that female mice exhibited early cardiac remodeling gene changes, all m6A machinery genes correlated significantly with LVEF in female mice after short-term diet exposure only; these relationships were abolished with a longer-term obesogenic diet. We speculate that cardiac m6A machinery may be regulated by different durations of obesogenic diet and differs according to sex, suggesting RNA methylation via m6A modification is dynamically involved in sex-based cardiac responses to diet.

Although this is the first study to describe sex-based cardiac differences in cardiac transcriptome and involvement of m6A RNA methylation system, induced by variable duration of diet-induced obesity, our study has several limitations. First, we cannot definitively demonstrate the cause and effect of RNA methylation systems in the development of obesity-induced cardiac changes. More detailed characterization of genome-wide m6A-sequencing analysis or manipulation of these systems with knockdown or overexpression will confirm their involvement in the pathophysiology of obesogenic stress-induced cardiac perturbations. Second, we have not fully characterized cardiac structural changes and possible associations with RNA methylation or transcriptomic systems, which would shed more light on their dynamic involvement from early cardiac remodeling and subsequent cardiac functional decline. Third, as our study predominantly focuses on elucidating gene changes, it is possible that further examination of protein changes will allow us to confirm the molecular drivers of these sex-based changes.

Our analysis identified pathways that may not immediately suggest a cardiometabolic link. However, for example, closer inspection of genes involved in the “cocaine addiction” pathway reveals that they are also involved in cardiac regulation: glutamate receptor subunit (GRIN2C) is involved in regulating calcium transport in heart tissues (94); activating transcription factor 4 (ATF4) is a transcription factor for redox regulators in cardiomyocytes (95). As most genes have multiple and context-dependent functions, it is unavoidable that seemingly unrelated pathways are identified. Enrichment analyses are also limited by the curation of current gene annotations and biases are likely as databases overlook unannotated genes and rely on traditionally male-based data. Different platforms also use varying ranking and statistical approaches. To mitigate some of these limitations, we have performed comprehensive analyses to include different databases and statistical methods with consistently robust results.

**Figure 10.**
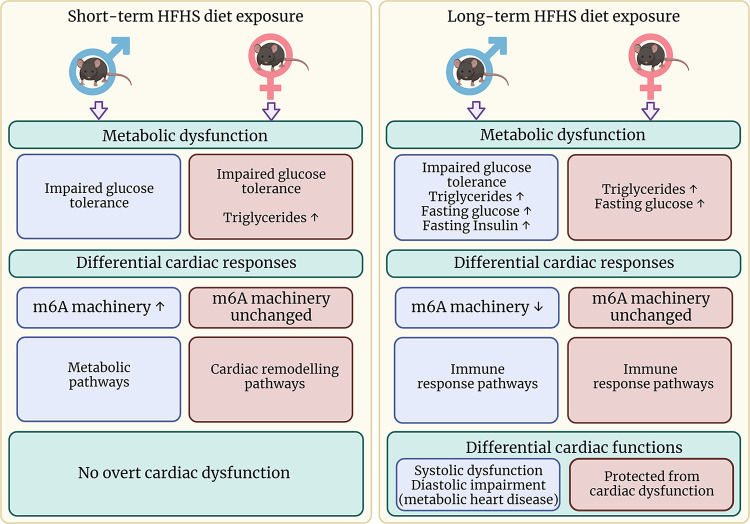
Summary of sex differences after short- and long-term exposure of male and female mice to high-fat/high-sucrose diet. Generated using Biorender.com.

In conclusion, our data (summarized in Fig. 10) provide novel insights into the sex-specific differences in cardiac transcriptome regulation in response to short- and long-term obesogenic stress. We demonstrate that protective cardiac remodeling soon after the start of an obesogenic diet may play a role in protecting females from developing cardiac dysfunction. RNA methylation machinery gene expression is regulated in a sex-dependent manner after diet exposure and correlates with cardiac function in an age- and sex-dependent manner, suggesting RNA methylation may play a role in sex dimorphism in diet-induced metabolic heart disease.

## DATA AVAILABILITY

Data is available at https://www.doi.org/10.5281/zenodo.8191388.

## SUPPLEMENTAL DATA

10.5281/zenodo.8191388 Supplemental material: https://doi.org/10.5281/zenodo.8191388.

## GRANTS

A.L.S. is supported by Heart Foundation of Australia Future Leader Fellowships Grants 101918 and 106025. D.T.M.N. is supported by the NSW Ministry of Health EMC Fellowship (Australia) and Heart Foundation of Australia Future Leader Fellowship Grant 104814. T.J.H is supported by NSW Ministry of Health Translational Research grant (held by A.L.S.). A.J.C. is supported by Vice Chancellor’s HDR Training Scholarship, University of Newcastle. C.K. is supported by Hunter Cancer Research Alliance PhD Scholarship for Translational Cancer Research. D.C. is supported by UNRSC Central Scholarship, University of Newcastle. This work was supported in part by NSW Ministry of Health Translational Research grant (to A.L.S.), NSW Ministry of Health Cardiovascular Research Capacity Program Early-Mid Career Researcher grant (to A.L.S. and D.T.M.N.), Department of Health and Aged Care Medical Research Future Fund Grant MRF2017053 (to A.L.S. and D.T.M.N.), and John Hunter Hospital Charitable Trust grants (to A.L.S., A.J.C., C.K., and D.C.).

## DISCLOSURES

No conflicts of interest, financial or otherwise, are declared by the authors.

## AUTHOR CONTRIBUTIONS

A.L.S. and D.T.M.N. conceived and designed research; A.J.C., C.K., D.C., and L.A.M. performed experiments; A.J.C. and L.A.M. analyzed data; A.J.C., A.L.S., and D.T.M.N. interpreted results of experiments; A.J.C. prepared figures; A.J.C., A.L.S., and D.T.M.N. drafted manuscript; A.J.C., T.J.H., L.B., A.J.B., A.L.S., and D.T.M.N. edited and revised manuscript; A.L.S. and D.T.M.N. approved final version of manuscript.
